# A late-lineage murine neutrophil precursor population exhibits dynamic changes during demand-adapted granulopoiesis

**DOI:** 10.1038/srep39804

**Published:** 2017-01-06

**Authors:** Min-Hyeok Kim, Dongchan Yang, Mirang Kim, Seon-Young Kim, Dongsup Kim, Suk-Jo Kang

**Affiliations:** 1 Department of Biological Sciences, Korea Advanced Institute of Science and Technology, Daejeon, 34141, Republic of Korea; 2 Department of Bio and Brain Engineering, Korea Advanced Institute of Science and Technology, Daejeon, 34141, Republic of Korea; 3 Medical Genomics Research Center, Korea Research Institute of Bioscience and Biotechnology, Daejeon, 34141, Republic of Korea

## Abstract

Homeostasis of neutrophils—the blood cells that respond first to infection and tissue injury—is critical for the regulation of immune responses and regulated through granulopoiesis, a multi-stage process by which neutrophils differentiate from hematopoietic stem cells. Granulopoiesis is a highly dynamic process and altered in certain clinical conditions, such as pathologic and iatrogenic neutropenia, described as demand-adapted granulopoiesis. The regulation of granulopoiesis under stress is not completely understood because studies of granulopoiesis dynamics have been hampered by technical limitations in defining neutrophil precursors. Here, we define a population of neutrophil precursor cells in the bone marrow with unprecedented purity, characterized by the lineage^−^CD11b^+^Ly6G^lo^Ly6B^int^CD115^−^, which we call NeuPs (Neutrophil Precursors). We demonstrated that NeuPs differentiate into mature and functional neutrophils both *in vitro* and *in vivo*. By analyzing the gene expression profiles of NeuPs, we also identified NeuP stage-specific genes and characterized patterns of gene regulation throughout granulopoiesis. Importantly, we found that NeuPs have the potential to proliferate, but the proliferation decreased in multiple different hematopoietic stress settings, indicating that proliferating NeuPs are poised at a critical step to regulate granulopoiesis. Our findings will facilitate understanding how the hematopoietic system maintains homeostasis and copes with the demands of granulopoiesis.

Neutrophils, the most abundant leukocytes in the blood, orchestrate immediate immune responses at an early time point in infection, serving to clear the pathogen and resolve acute inflammatory responses and tissue injury^1^,^2^,^3^,^4^,^5^. The duration and magnitude of inflammation are affected by the transit of neutrophil precursors to mature neutrophils, the migration of neutrophils into the infected or injured area, death and clearance of dead neutrophils. The loss of neutrophils is balanced by regeneration processes that serve to maintain neutrophil homeostasis.

Mature neutrophils are non-dividing cells and circulate in the blood for few hours to days^6^,^7^,^8^,^9^. Thus, maintaining circulating neutrophil numbers requires the production and mobilization of billions of neutrophils per kilogram body weight per day in humans^10^,^11^,^12^. Neutrophils develop in the bone marrow from hematopoietic stem cells through a process involving multiple successive stages of neutrophil precursors, including common myeloid progenitors (CMPs) and granulocyte-macrophage progenitors (GMPs). Neutrophil precursor populations were initially identified based on morphological and cellular component analysis^13^. Neutrophil precursors mature from proliferating precursors (myeloblasts, promyelocytes, and myelocytes) via post-mitotic precursors (metamyelocytes and band cells), finally becoming mature segmented neutrophils that are mobilized from the bone marrow into the blood circulation. The maturation process takes about 10–12 days^14^ and is paralleled by the acquisition of distinct granule proteins at different precursor stages^15^.

Certain clinical conditions lead to neutropenia, which triggers demand-adapted granulopoiesis^16^,^17^. Systemically disseminated infection can lead to massive death of neutrophils, which turns on emergency granulopoiesis. Non-infectious settings such as myeloablation due to chemotherapy or ionizing radiation, allergic responses, or autoimmune disorders can also cause neutropenia and activate a different type of demand-adapted granulopoiesis called reactive granulopoiesis^17^. Exquisite regulation of these processes is critical for resolving the clinical condition. It has been reported that the levels of granulopoietic cytokines, including granulocyte colony-stimulating factor (G-CSF), are upregulated in human patients suffering from anemia, leukemia, or infections^18^,^19^,^20^. However, the relationship between serum G-CSF levels and neutrophil numbers is complex and highly dynamic, as neutrophils themselves are major contributors to G-CSF clearance by a G-CSF receptor dependent mechanism^21^,^22^.

G-CSF is an essential granulopoietic growth factor. In steady state, signaling via G-CSF and its receptor, G-CSFR, maintains circulating neutrophil numbers by enhancing the proliferation and differentiation of precursors^12^,^23^,^24^,^25^,^26^,^27^,^28^,^29^,^30^, as well as by increasing the survival^29^,^31^,^32^ and mobilization of mature neutrophils^25^,^27^,^28^,^33^,^34^,^35^. Exogenously added G-CSF shortens the mean cycle time of mitotic cells and the transit time of the post-mitotic pool, but does not affect the half-life of circulating neutrophils *in vivo*^12^,^27^. It has also been found that G-CSF is crucial for emergency granulopoiesis caused by infection with *Listeria monocytogenes* (but not *Candida albicans*)^28^,^29^,^36^,^37^. Recent studies have demonstrated that Toll-like receptor 4 (TLR4) expressed on endothelial cells, but not on hematopoietic cells, is responsible for emergency granulopoiesis^38^,^39^.

Previous studies have reported myeloid transcription factors that regulate granulopoiesis under steady state and emergency conditions. At steady state, PU.1 plays a pleiotropic role in hematopoiesis^40^,^41^, and its expression level determines macrophage versus neutrophil lineage^42^. C/EBPα plays a critical role in the transition from CMPs to GMPs, by suppressing the expression of *c-Myc*^43^,^44^,^45^. Maturation beyond the promyelocyte stage requires GFI1, C/EBPε, and LEF1^46^,^47^,^48^,^49^. GFI1 and C/EBPε are required for formation of neutrophil secondary granules^23^,^46^,^50^. Under emergency conditions, however, C/EBPβ plays a crucial role in regulating granulopoiesis^51^,^52^. STAT3 links G-CSF signaling to the expression of C/EBPβ, thereby enhancing neutrophil production^53^,^54^ and mobilization^54^.

Understanding the mechanisms underlying granulopoiesis is essential for resolving clinical conditions that induce aberrant neutrophil numbers, and requires isolation of precursors with high purity and in-depth genomic analysis of the developmental stage. Previous isolation protocols were based on density gradient centrifugation^55^,^56^, which showed enrichment of neutrophil precursors to a limited purity. Multicolor flow cytometry analysis of surface molecules such as CD15, CD16, CD24, CD33, and CD49d in humans has been used to discern neutrophil precursors more accurately^49^,^57^. In contrast, murine neutrophil precursors have been distinguished by differential expression of CD11b and Gr-1^6^,^54^,^58^,^59^. However, RB6–8C5, a monoclonal antibody for Gr-1, recognizes both Ly6C and Ly6G^44^, an overlapping specificity that has confounded neutrophil precursor analysis because Ly6C is expressed on many types of myeloid cells, including monocytes.

Here, we developed a novel scheme to isolate murine bone marrow neutrophil precursors based on the expression of Ly6B and CD115. Ly6B is a Ly6 family protein that can be detected by the 7/4 clone antibody and is expressed on neutrophils, inflammatory monocytes, and activated macrophages^60^. We confirmed that the isolated neutrophil precursors have the potential to differentiate into functional neutrophils both *in vitro* and *in vivo*. By analyzing the gene expression profile of NeuPs, which further re-confirmed the neutrophil lineage of NeuPs, we determined and functionally catalogued NeuP-specific genes compared to those of GMPs, neutrophils, and monocytes. Importantly, we found that NeuPs are proliferative precursor cells and the proliferation of NeuPs decreased during demand-adapted granulopoiesis, demarcating the NeuP stage as a critical common regulatory point under hematopoietic stress.

## Results

### Identification of CD11b^+^Ly6G^lo^Ly6B^int^CD115^−^ neutrophil precursor cells in the BM

**Figure 1:**
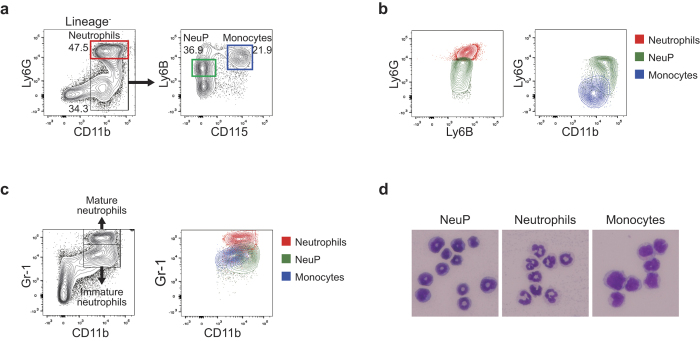
Identification and characterization of NeuPs. (**a**) Multicolor flow cytometry analysis of murine BM populations. Murine BM cells from femur and tibia were labeled for lineage (CD3ε, CD19, NK1.1, and B220), CD11b, Ly6G, Ly6B, and CD115 and analyzed by flow cytometry. (**b**) Expression of Ly6B, Ly6G, and CD11b among neutrophils, monocytes and NeuPs, analyzed by flow cytometry. (**c**) Comparison of the conventional neutrophil precursor analysis based on Gr-1 versus CD11b (left panel) with our NeuP analysis. Neutrophil progenitors defined by the conventional analysis included monocytes (right panel). (**d**) Micrographs of sorted NeuPs, neutrophils, and monocytes stained with May-Grünwald-Giemsa solution.

To define murine neutrophil precursors in the BM, we analyzed lineage-negative (CD3ε^−^CD19^−^NK1.1^−^B220^−^) BM cells for expression of CD11b, CD115, Ly6B, and Ly6G by flow cytometry (Fig. 1a). Neutrophils expressing Ly6G at high levels in the lineage-negative populations were excluded and the remaining CD11b^+^ cells were further analyzed based on the expression level of CD115 and Ly6B. We observed that Ly6B^+^ cells could be divided into two distinct populations based on their CD115 expression level. The Ly6B^hi^CD115^+^ population were inflammatory monocytes, as previously reported^61^. The unknown Ly6B^int^CD115^−^ population expressed Ly6B at a level comparable to that of neutrophils, but expressed Ly6G at lower levels (Fig. 1b).

We compared results obtained using our method with previous analyses of immature and mature neutrophils based on Gr-1 and CD11b expression^6^,^54^,^58^,^59^ (Fig. 1c). The Gr-1^hi^ population consists of mature neutrophils while Gr-1^int^CD11b^+^ cells are a population of immature neutrophil precursors. We observed that the Ly6B^int^CD115^−^ population was found within Gr-1^int^CD11b^+^ immature neutrophils. Therefore, we suspected that the Ly6B^int^CD115^−^ cells are neutrophil precursor cells and termed them NeuPs (Neutrophil Precursors). Remarkably, we noted that Gr-1^int^CD11b^+^ cells were not pure immature neutrophil precursors, but included inflammatory monocytes (Fig. 1c). Similarly, the gating scheme shown in a recent study attempting to dissect neutrophil developmental stages based on the differential expression of c-kit and Ly6G^52^ was also unable to prevent CD115^+^ monocyte contamination in each precursor population (Supplementary Fig. S1).

We sorted NeuPs from BM using multicolor flow cytometry (Supplementary Fig. S2). Cytological analysis with May-Grünwald-Giemsa staining of neutrophils, monocytes, and NeuPs isolated from BM revealed that NeuPs are morphologically distinct from neutrophils and monocytes (Fig. 1d). NeuPs had either ring-shaped or peanut-shaped nuclei, whereas neutrophils had segmented lobular nuclei. Murine neutrophil or macrophage precursor populations in the BM have been reported to have ring-shaped nuclei^62^. The size and granularity of NeuPs were similar to those of neutrophils, but distinct from those of eosinophils (Supplementary Fig. S3a). Little or no expression of the basophil markers, DX5 and Mar-1, or the eosinophil marker, Siglec-F, was detected on NeuPs (Supplementary Fig. S3b). NeuPs express CD24, CD44, and CD172 at high level similar to BM neutrophils and monocytes (Supplementary Fig. S3c).

Taken together, these findings demonstrate the successful development of a novel flow cytometry scheme for isolating a neutrophil precursor population with the lineage CD11b^+^Ly6G^lo^Ly6B^int^CD115^−^, with minimal contamination by other types of myeloid cells.

### NeuPs effectively differentiate into a neutrophil population *in vitro* and *in vivo*

**Figure 2:**
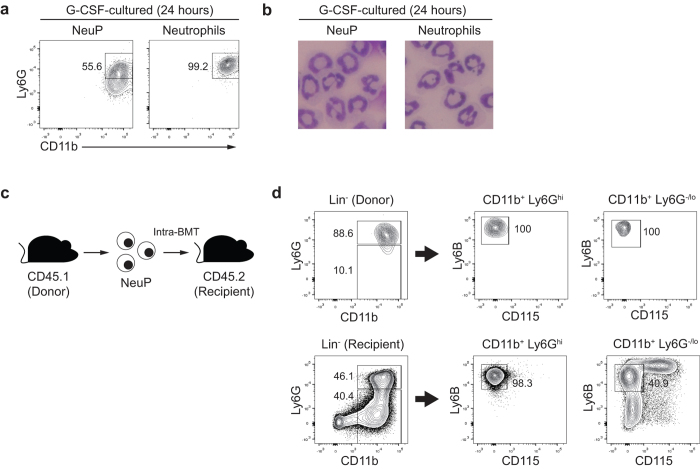
NeuPs give rise to neutrophils. (**a**) Flow cytometry analysis of NeuPs and neutrophils that were sorted and cultured with G-CSF. (**b**) Cytological analysis of NeuPs and neutrophils cultured as in (**a**) and stained with May-Grünwald-Giemsa solution. (**c**) Experimental scheme for analysis of NeuP differentiation *in vivo*. NeuPs were sorted from CD45.1 congenic mice, transferred to CD45.2 recipient mice through intra-BM transfer, and analyzed in BM 3 days after transfer. (**d**) Expression of Ly6G (neutrophil differentiation) and CD115 (monocyte differentiation) in donor (upper three panels) and recipient cells (lower three panels), assessed by flow cytometry. Data are representative of at least two independent experiments (N = 3 mice/group).

To firmly establish that NeuPs have the potential to differentiate into neutrophils, we sorted NeuPs or mature neutrophils from BM and cultured them in the presence of G-CSF. After 24 hours of culture, NeuPs expressed the mature neutrophil marker, Ly6G (Fig. 2a). Furthermore, the ring-shaped nuclei of NeuPs adopted the very dynamic, lobular form of nuclei of mature neutrophils (Fig. 2b). These data indicate that NeuPs did in fact differentiate into neutrophils. We further validated the neutrophil-differentiation potential of NeuPs *in vivo.* Since we could not detect transferred NeuPs after intravenous injection (data not shown), we injected NeuPs directly into the BM, which is expected to provide a niche for neutrophil differentiation (Fig. 2c). NeuPs sorted from CD45.1 mice were transferred to CD45.2 congenic mice, and NeuP-derived cells were detected and analyzed by staining for congenic markers. Most transferred NeuPs displayed high levels of Ly6G expression and no other lineage markers, such as the monocyte marker CD115 (Fig. 2d), indicating that NeuPs give rise only to neutrophils *in vivo*. Thus, these data further confirm that NeuPs are *bona fide* neutrophil precursors.

### Functional analysis of NeuPs and NeuP-derived neutrophils

**Figure 3:**
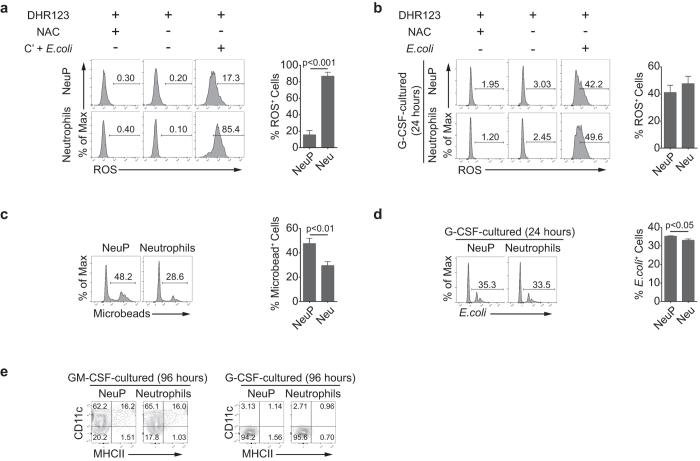
NeuP-derived neutrophils are functionally equivalent to neutrophils. (**a**) ROS production by NeuPs and neutrophils that were sorted and stimulated with or without complement and *E. coli*. ROS-producing cells were detected using dihydrorhodamine 123 (DHR123) and analyzed by flow cytometry. N-acetylcysteine (NAC) was treated while staining with DHR123 to show the negative staining. Data are representative of two independent experiments (mean ± SD of N = 3 mice/group). (**b**) ROS production by G-CSF-cultured NeuPs and neutrophils that were stimulated with or without *E. coli*. ROS-producing cells were detected as in (**a**). (**c**) The *ex vivo* phagocytic ability of NeuPs and neutrophils. NeuPs and neutrophils were sorted and incubated with FITC-conjugated microbeads. Cells taking up the beads were analyzed by flow cytometry. (**d**) The phagocytic ability of G-CSF–cultured NeuPs and neutrophils. Cells were incubated with tdTomato-expressing *E. coli* and analyzed by flow cytometry. (**b–d**) Data are representative of three independent experiments (mean ± SD of N = 3 mice/group). (**e**) Expression of DC markers, CD11c and class II, in NeuPs and neutrophils cultured with GM-CSF for 96 hours, assessed by flow cytometry (left panel). Expression of CD11c and class II in NeuPs and neutrophils cultured with G-CSF for 96 hours, assessed by flow cytometry (right panel). Data are representative of two independent experiments.

Next, we evaluated function of NeuPs and determined whether NeuPs differentiate into functional neutrophils by examining reactive oxygen species (ROS) production and phagocytic activity. Neutrophils can be induced to produce ROS by stimulating with complement and bacterial stimulation^2^,^63^,^64^. We evaluated *ex vivo* ROS production ability of NeuPs and neutrophils that were freshly isolated from BM under the treatment of complement and *Escherichia coli*. About 80% of mature neutrophils actively produced ROS under the stimulation, whereas around 15% of NeuPs produced ROS at lower levels (Fig. 3a). We also treated G-CSF–cultured neutrophils and NeuP-derived neutrophils with or without *E. coli*. NeuP-derived neutrophils become as potent ROS-producing cells as neutrophils under bacterial stimulation (Fig. 3b). In contrast to ROS production, NeuPs displayed higher capacity to phagocytose fluorescein-conjugated beads than neutrophils *ex vivo* (Fig. 3c). After *in vitro* differentiation, about 35% of NeuP-derived neutrophils engulfed *E. coli*, a percentage slightly greater than that for mature neutrophils (Fig. 3d). These results confirm that NeuPs are capable of differentiating into fully functional neutrophils.

Previous reports have shown that GM-CSF can induce neutrophils to adopt dendritic cell (DC)-like characteristics^65^,^66^. To determine whether NeuPs could be similarly induced to display DC-like properties, we treated them with GM-CSF and examined expression of CD11c, a marker of DCs. After 4 days in culture with GM-CSF, about 80% of neutrophils and NeuPs were CD11c^+^ (Fig. 3e). Some even expressed MHC class II molecules, indicating that GM-CSF can drive DC-like differentiation of NeuPs in a manner similar to its effects on neutrophils. However, G-CSF had no such effect on neutrophils or NeuPs (Fig. 3e). Interestingly, overall survival was better with GM-CSF than with G-CSF: a number of cells still survived after 6 days of GM-CSF-supplemented culture (data not shown).

### Transcriptome analysis of NeuPs

**Figure 4:**
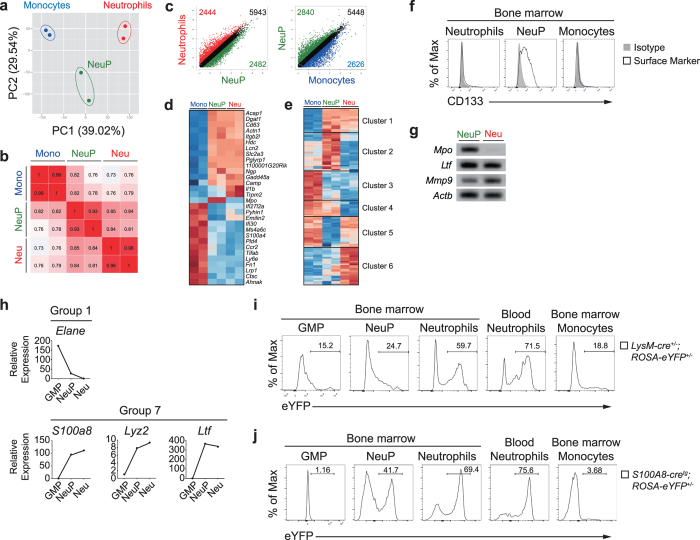
Transcriptome analysis of NeuPs. (**a**) PCA analysis of gene expression profiles of monocytes, NeuPs, and neutrophils. Numbers in parenthesis indicate the percentage of the total variance represented by the axis. (**b**) Correlation matrix of monocytes, NeuPs, and neutrophils based on all available genes. Numbers in plots indicate correlation coefficient values. (**c**) Diversity of gene expression in monocytes, NeuPs, and neutrophils. Numbers in plots indicate probes with a minimum change of 2-fold in expression. Cell types are color-coded. (**d**) Heat map and hierarchical clustering of mRNA transcripts of DEGs determined by Cuffdiff (FDR < 0.05). (**e**) Heat map of genes encoding transcription factors whose expression showed more than a 3-fold change between cell types and less than a 2-fold difference between replicates. Pearson distance is used as a distance function. Genes were clustered into six groups and are listed in Table 1. (**f**) Expression level of CD133, novel candidate for NeuP specific surface marker, on NeuPs, neutrophils and monocytes were detected with FACS. (**g**) RT-PCR of genes for granule markers in NeuPs and neutrophils. This is a processed and cropped version. The original full-length gel image is presented in the Supplementary Figure S9. (**h**) Expression of neutrophil lineage genes (*Elane, S100a8, Lyz2*, and *Ltf*) among GMPs, NeuPs, and neutrophils. Relative expression compared to neutrophils is shown. (**i-j**) EYFP labeling of GMPs, NeuPs, neutrophils, and monocytes based on *Lyz2* expression by using *Lyz2-cre; ROSA-eYFP* mice (**i**) or on *S100a8* expression by using *S100A8-cre; ROSA-eYFP* mice (**j**). Percentage of eYFP-expressing cells, determined in comparison to WT mice, is shown. Data are representative of two independent experiments.

To further substantiate the identity of NeuPs and determine the NeuP-stage specific genes, we performed genome-wide expression profiling through RNA-sequencing of sorted NeuPs, monocytes and neutrophils, and evaluated the relationships among the three populations. A principal component analysis (PCA) of all genes with two major principal components showed that NeuPs were separated from neutrophils and monocytes by a substantial distance (Fig. 4a). Pearson correlation coefficients between transcription profiles of each sample were calculated and displayed as a heat map. Correlation coefficients between NeuPs and neutrophils ranged from 0.81 to 0.85, a range higher relative to that between NeuPs and monocytes (0.76–0.82), indicating that NeuPs are more closely related to neutrophils than to monocytes (Fig. 4b). A scatter plot analysis revealed that, among the ~11,000 mRNA transcripts analyzed, 2,482 and 2,840 genes were expressed higher in NeuPs by more than a 2-fold difference in expression compared to neutrophils and monocytes, respectively (Fig. 4c). A heat map analysis of differentially expressed genes (DEG) with a false discovery rate (FDR) of less than 0.05 revealed that NeuPs clustered more closely with neutrophils than monocytes (Fig. 4d). We also determined potentially NeuP stage-specific transcription factors by analyzing the expression pattern of transcription factors among the three populations (Fig. 4e; and Table 1). These include transcription factors previously known to be critical for neutrophil development, such as C/EBPε^23^,^50^, GFI1^46^,^49^, HLX^67^, and MYB^55^. Some identified genes, such as *Hmgb3, Ssrp1* and *Foxm1*, have not been previously associated with neutrophil development and may be novel NeuP-specific transcription factors. *Foxm1* was known to regulate expression of *Prom1* gene (also known as CD133)^68^. Prom1 (CD133) expression was exclusively high on NeuPs in our transcriptome analysis, so we examined whether CD133 can be used as a novel surface marker for NeuPs. CD133 was distinctly detected on NeuPs compared to neutrophils and monocytes in the flow cytometric analysis (Fig. 4f). Overall, NeuPs showed a closer relationship to neutrophils than monocytes, but exhibited unique gene expression profiles, indicating the dynamic nature of the neutrophil development process. To verify RNA-seq results and further specify the stage of NeuPs, we confirmed the expression levels of three major granule genes—*Mpo* (myeloperoxidase, primary granule marker, enriched in promyelocytes), *Ltf* (lactotransferrin, secondary granule marker) and *Mmp9* (matrix metallopeptidase 9, tertiary granule marker, enriched in neutrophils)^15^—by RT-PCR (Fig. 4g). NeuPs expressed *Mpo* and *Ltf* at high levels and showed lower expression of *Mmp9* compared to neutrophils. Consistent with their morphology (Fig. 1d), transcriptome profiling also positions NeuPs between promyelocytes and neutrophils.

To explore the dynamics of gene expression and categorize genes according to the expression patterns during granulopoiesis, we combined our RNA-seq data with GMP and neutrophil gene expression data obtained from the ImmGen database (Supplementary Fig. S4). A total of 9,158 genes were found in common between the two datasets and were used for further analysis. We calculated relative gene expressions normalized to that of neutrophils and categorized the genes into nine gene expression profile groups based on expression patterns along the transition from GMPs to NeuPs and neutrophils (Supplementary Table S1).

A gene ontology (GO) analysis of genes in groups 1–8 showing altered gene expression was performed using DAVID (Supplementary Fig. S5; and Supplementary Table S2). Expression of genes in groups 1–3 gradually decreased, whereas that of genes in groups 5–7 increased, along the course of neutrophil maturation. Genes in groups 4 and 8 were up-regulated and down-regulated, respectively, only in NeuPs. Overall, genes in groups 1–4 are mainly involved in cell cycle control and metabolic process related to the synthesis of genetic materials or cellular components. Groups 1–3 genes included those encoding transcription and translation machinery components, such as transcription factors, translation initiators, and aminoacyl tRNA synthetases. Although catalogued as metabolic genes, each group contained distinct gene sets. Group 1 included metabolic genes for tRNA synthesis and protein modification, whereas group 2 included genes for carbohydrate, lipid and protein anabolic and catabolic processes, and group 3 genes are involved in DNA replication and repair. Notably, group 4 included genes involved in stimulating cell division and coordinating cell cycle phases, such as checkpoint and chromatin segregation, implying that control of cell division is critical task at the NeuP stage. In contrast, genes in groups 5–8 are related to development of the immune system and immune responses. Notably, groups 5 and 6 included genes that function in innate immune signaling, such as *Tlr* (-*2, -4, -6* and *-8*), *Myd88, Ddx58, Dhx58, Nlrp3*, and *Oas* family genes. Expression of cytokine genes, such as *Il1b* and *Il15*, was also upregulated as neutrophils mature. Collectively, the results of our GO term analysis reveal that, during maturation, neutrophil precursors come to express genes for immune-effector functions, concomitantly losing expression of genes for metabolism and proliferation, a transition that is highlighted at the NeuP stage.

Because increasing evidence suggests that epigenetic regulation plays a critical role in controlling hematopoiesis^69^,^70^, we selected and analyzed DEGs that serve as epigenetic regulators. Epigenetic regulators were detected in five groups (groups 1, 2, 4, 6, and 7). Each group included distinct types of chromatin remodelers and epigenetic regulators involved in histone modifications (Supplementary Fig. S6). This analysis suggests the possibility that stages of granulopoiesis are determined by distinct types of epigenetic regulators; further investigation will be required to corroborate this notion.

Previous data showed that mice deficient of JDP2, a transcription regulator of the AP-1 family, exhibited accumulation of immature neutrophils and primary granule genes. JDP2 suppresses expression of ATF3 which negatively regulates neutrophil differentiation via inhibiting histone acetylation at the promoter of ATF3^71^. It was shown that JDP2 recruits HDAC families (HDAC1, 2–6, and 10) to the ATF3 promoter^72^. In our analysis, *Jdp2* was detected as a neutrophil lineage specific transcription factor compared to monocytes (Table 1, cluster 1) and within group 7 (Supplementary Table S1) in which gene expression is highly upregulated in NeuPs and neutrophils compared to GMPs. In contrast, HDAC2 and 6, found within group 2, were highly expressed at the GMP stage and then significantly decreased at the NeuP stage. This inverse correlation in expression pattern of JDP2 and HDAC family proteins may explain the potential role of JDP2 in regulating neutrophil development at early stages of granulopoiesis.

Many genetically modified strains of mice that harbor genes encoding fluorescence reporter proteins have been used to track down specific lineages. Similarly, mice expressing cre recombinase together with a diphtheria toxin or its receptor have been developed in order to trace or conditionally delete cells of a target lineage^73^. Gene loci encoding *S100A8*^74^,^75^, *Lyz2*^76^,^77^, *Ltf*^78^, *Elane*^79^, and *Ly6g*^80^ have been used for targeting neutrophils, although some other types of myeloid cells are labeled to a certain degree. For example, monocytes and macrophages are also labeled in *Lyz2*(*LysM*)*-cre* mice. Previously, it was shown that the labeling efficiencies vary among the neutrophil specific cre strains: *S100A8*(*MRP8*)*-cre* mice showed over 80% recombination efficiency in neutrophils whereas *Lyz2-cre and GE* (*Elane*)*-cre* mice showed 50–70% recombination rates^73^. We examined expression of the neutrophil specific genes in GMPs, NeuPs, and neutrophils from our analysis (Fig. 4h). *Elane*, found in group 1, is expressed 200-fold and 20-fold higher in GMPs and NeuPs than neutrophils, respectively. Expression of *S100A8, Lyz2*, and *Ltf*, categorized as group 7 genes, is much higher in NeuPs and neutrophils than in GMPs. NeuPs showed around 100-, 8-, 300-fold increase in expression of *S100A8, Lyz2*, and *Ltf* than GMPs, respectively. However, the three genes are expressed similarly between NeuPs and neutrophils. Thus, we suspected that NeuPs could be marked by a cre recombinase that is expressed under the control of *S100A8, Lyz2* or *Ltf*. To test the possibility, we crossed *Lyz2-cre* and *S100A8-cre* mice with *ROSA-eYFP* mice in which a *loxP*-flanked transcription STOP sequence is followed by an eYFP-encoding cassette at the *ROSA* locus^81^ and traced the history of *Lyz2* and *S100A8* expression by eYFP expression. In *Lyz2-cre*^+/−^*; ROSA-eYFP*^+/−^ mice, about 25% of NeuPs expressed eYFP (Fig. 4i). In accordance with *Lyz2* expression that is higher in NeuPs than in GMPs (Fig. 4h), eYFP-expressing cells were around 10% more abundant in NeuPs compared to GMPs (Fig. 4i). Importantly, higher percentage of neutrophils in BM and blood were labeled than NeuPs. Furthermore, 40% of NeuPs and about 70% of neutrophils were eYFP-positive in *S100A8-cre; ROSA-eYFP*^+/−^ mice, yet little GMPs were labeled by eYFP (Fig. 4j). These results corroborate that NeuPs precede neutrophils in development.

### NeuPs have the proliferative potential, which is modulated during demand-adapted granulopoiesis

**Figure 5:**
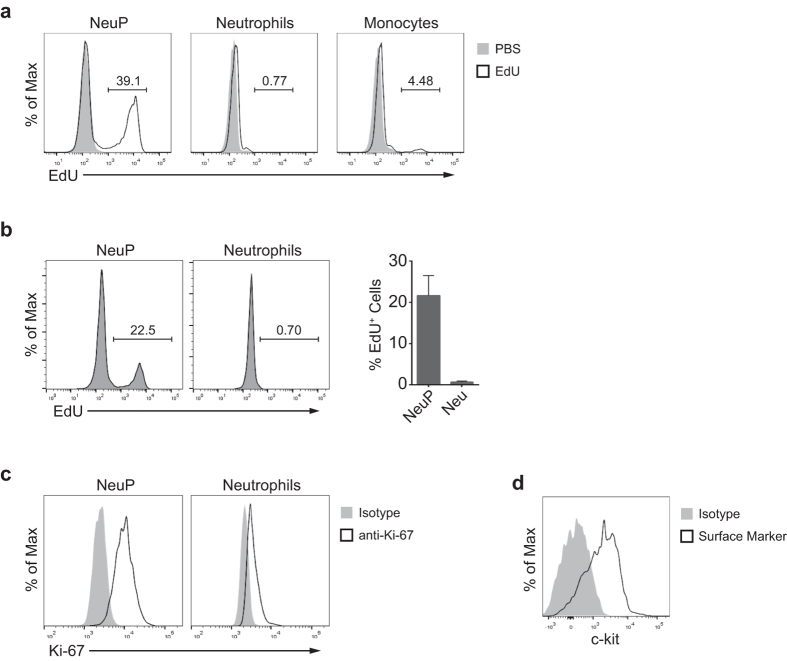
NeuPs are proliferative precursor cells. (**a**) Proliferation of NeuPs, neutrophils and monocytes, measured as EdU incorporation by flow cytometry. Each cell type was sorted from mice that were injected with EdU 2 hours prior to sacrifice, and stained for EdU. (**b**) NeuPs and neutrophils were sorted from the bone marrow of wild type mice and cultured for 2 hours in the medium supplemented with 10 μM EdU. EdU incorporation was analyzed by flow cytometry. Data are representative of two independent experiments (mean ± SD of N = 3). (**c**) Expression of Ki-67 of NeuPs and neutrophils, analyzed by flow cytometry. (**d**) Expression of c-kit on NeuPs.

Our gene expression profiling of NeuPs revealed that genes regulating cell cycle are enriched in NeuPs (Supplementary Fig. S5) and that NeuPs are developmentally located between proliferating promyelocytes and non-proliferating neutrophils (Fig. 4g). Therefore, we examined whether NeuPs have the proliferative potential by incorporation of EdU (5-ethynyl-2′-deoxyuridine) in NeuPs, an analog of thymidine, as a marker of DNA synthesis. EdU was injected intravenously into mice 2 hours before harvesting BM cells. Compared to neutrophil and inflammatory monocyte populations, which had few EdU^+^ cells, approximately 40% of NeuPs were EdU^+^, indicating that NeuPs are actively proliferating cells (Fig. 5a). Since the EdU^+^ cells may have acquired EdU before NeuP stage, we first sorted NeuPs and then immediately labelled *in vitro* the proliferating NeuPs by incubating with EdU (Fig. 5b). We observed EdU-stained NeuPs in this setting, confirming NeuP’s intrinsic proliferative potential. Similar results were obtained by staining for Ki-67, another marker of proliferating cells (Fig. 5c). As observed in proliferating early hematogenic precursors and neutrophil precursor cells^52^,^56^, NeuPs also expressed c-kit (Fig. 5d).

**Figure 6:**
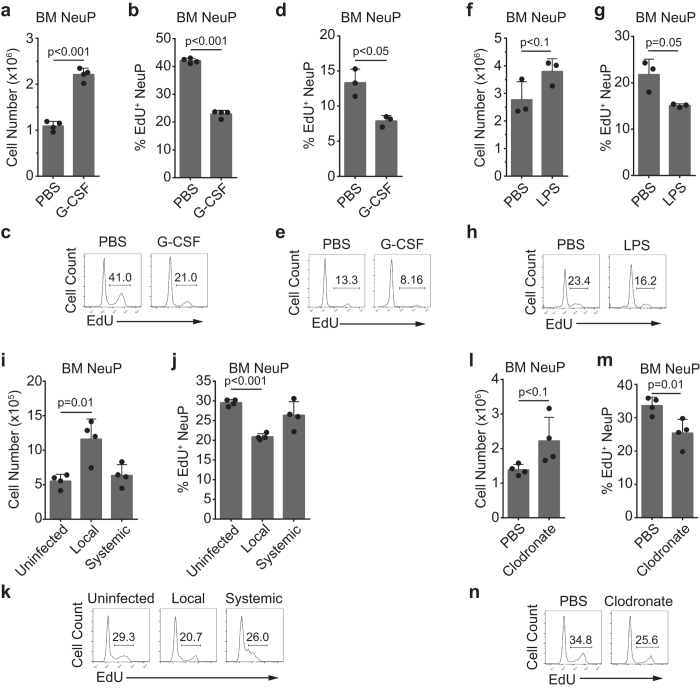
NeuP proliferation decreases during demand-adapted granulopoiesis. (**a–h**) Twenty-four hours prior to sacrifice, mice were injected with PBS (control) or G-CSF (2.5 μg) (**a–e**), or with PBS or LPS (20 μg) (**f–h**). (**i–n**) Forty-eight hours before sacrifice, mice were injected with PBS (control) via the tail vein or with *Listeria* through footpads (10^4^ cfu; local infection) or tail veins (10^3^ cfu; systemic infection) (**i-k**) or were injected with PBS- or clodronate-liposomes (1 mg) (**l–n**). (**a–c, f–n**) Mice were injected with EdU (0.5 mg) 2 hours before sacrifice. (**d,e**) NeuPs were sorted from the bone marrow of the PBS- or G-CSF-injected mice and cultured for 2 hours in the medium supplemented with 10 μM EdU. EdU incorporation was analyzed by flow cytometry. Bone marrow cells were analyzed for cell numbers and proliferation. Graphs of NeuP cell numbers (**a,f,i,l**) and percentage of EdU^+^ NeuPs (**b,d,g,j,m**) are shown. NeuP proliferation, assessed by measuring EdU incorporation, is shown as flow cytometry plots (**c,e,h,k,n**). The significance of differences between stimulated and control groups was analyzed using Student’s *t*-test. Data are representative of at least two independent experiments (mean ± SD of N = 3 to 4 mice/group).

Stress conditions that result in a severe reduction in neutrophils can trigger demand-adapted granulopoiesis^16^,^17^. Our isolation of highly pure neutrophil precursors enabled us to track dynamic alterations in neutrophil precursors in terms of number and proliferation potential during emergency granulopoiesis. Injection of G-CSF or LPS, mimicking emergency conditions, acutely enhances granulopoiesis^38^. Accordingly, we administered G-CSF to mice and examined NeuPs in bone marrow 24 hours later. G-CSF treatment doubled the number of bone marrow-resident NeuPs (Fig. 6a). No significant changes in the number of whole bone marrow cells or neutrophils were detected within this period (Supplementary Fig. S7a). Notably, the number of EdU^+^ proliferating cells significantly decreased after G-CSF treatment (Fig. 6b,c). Next, we analyzed *in vitro* proliferation of NeuPs sorted from the G-CSF- or PBS-treated mice and observed significantly less EdU^+^ NeuPs from G-CSF-stimulated mice, compared to PBS-treated control mice (Fig. 6d,e). This indicates that G-CSF-stimulation decreased the proliferative potential of the cells at the NeuP stage. Similarly, systemic LPS stimulation (intravenous injection) reduced the percentage of proliferating NeuPs 1 day after LPS stimulation (Fig. 6g,h), but we found an increase, but not significant, of bone marrow NeuPs at this time point (Fig. 6f). Of note, the number of whole bone marrow cells and neutrophils significantly decreased in LPS-treated mice compared with control mice (Supplementary Fig. S7b).

Next, we examined whether local and systemic bacterial infection resulted in different NeuP dynamics. Footpad injection of *Listeria* induces local infection, whereas intravenous injection leads to systemic infection^82^,^83^. NeuPs significantly increased in the bone marrow 48 hours after local infection, and the proportion of proliferating cells substantially decreased (Fig. 6i–k). However, NeuP cell numbers and the fraction of proliferating NeuPs did not change with systemic infection (Fig. 6i,j), but interestingly, the degree of EdU staining was decreased (Fig. 6k). The number of total bone marrow cells and neutrophils decreased in both cases, but more severely in systemic infection (Supplementary Fig. S7c). These results indicate that the behavior of neutrophil precursors varies dramatically depending on the type of infection.

We observed an increase in blood neutrophil numbers after ablation of monocytes/macrophages by treatment with clodronate-liposomes (data not shown). Thus, we wondered whether a non-infectious condition that might perturb the local bone marrow niche would affect granulopoiesis. To test the possibility, we treated mice with clodronate- or PBS (control)-liposomes and analyzed NeuPs. Clodronate-liposomes depleted circulating monocytes without significantly decreasing the number of total bone marrow cells or neutrophils (Supplementary Fig. S7d). Bone marrow-resident NeuP numbers trended slightly higher, although this difference was not significant (Fig. 6l). The EdU^+^ fraction of NeuPs, however, significantly decreased (Fig. 6m,n). Collectively, these results confirm that various conditions can trigger granulopoiesis and the changes in NeuPs are not uniform, suggesting a dynamic response.

**Figure 7:**
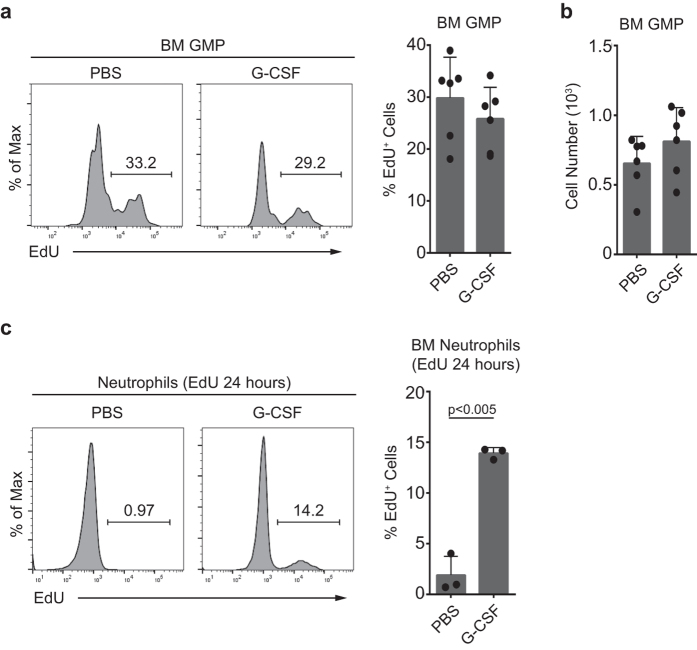
Accelerated differentiation during demand-adapted granulopoiesis. (**a,b**) Twenty-four hours prior to sacrifice, mice were injected with PBS (control) or G-CSF (2.5 μg) and bone marrow GMPs were analyzed for (**a**) proliferation and (**b**) cell numbers. Mice were injected with EdU (0.5 mg) 2 hours prior to sacrifice, and bone marrow cells stained with EdU were analyzed by flow cytometry. Graphs show the pooled data of two independent experiments with three biological replicates (mean ± SD of N = 6 mice/group). (**c**) Twenty-four hours prior to sacrifice, mice were injected with PBS (control) or G-CSF (2.5 μg) plus EdU (0.5 mg). Mice were sacrificed and EdU incorporation into neutrophils was analyzed by flow cytometry. Data are representative of two independent experiments (mean ± SD of N = 3 mice/group).

The increased number of NeuPs under demand-adapted granulopoiesis may be due to the increase of influx from the upstream precursors. However, GMP did not show any difference both in cell numbers and EdU incorporation under G-CSF stimulation, compared to PBS-treated mice (Fig. 7a,b). Next, we examined whether the differentiation kinetics has changed. We performed a pulse-chase experiment with EdU by injecting EdU along with G-CSF or PBS treatment and let EdU-labelled precursors differentiate into neutrophils for 24 hours, rather than 2 hours, and compared the percentage of EdU-labelled neutrophils. We could see increase of EdU-incorporated neutrophil populations with G-CSF-, but not with PBS-treatment (Fig. 7c). This result indicates that neutrophil differentiation has been accelerated by G-CSF stimulation, which may contribute to the accumulation of NeuPs in G-CSF-stimulated mice.

## Discussion

Here, we demonstrated that Ly6B, in combination with CD115, can be used to isolate and analyze neutrophil precursors, NeuPs, from bone marrow. Our results from flow cytometry, morphological analysis, gene expression profiling, *in vitro* culture, and *in vivo* transfer experiments provide convincing evidence that NeuPs are a population with the potential to proliferate in response to various stimuli and differentiate into functional neutrophils.

A transcriptome analysis of NeuPs revealed that this population is more closely related to neutrophils than monocytes. However, the gene expression profile of NeuPs is quite distinct from both, indicating that neutrophils undergo highly dynamic changes in gene expression during the maturation process. Combining microarray data for GMPs and neutrophils in the ImmGen database with our RNA-seq data, we determined stage-specific genes and functionally categorized gene expression patterns throughout granulopoiesis. This analysis identified many novel candidate genes, including transcription factors, that were previously uncharacterized in the context of neutrophil development and we are actively investigating their roles.

The importance of epigenetic regulation is an emergent theme in neutrophil development^69^,^70^,^71^,^84^. However, epigenetic factors that regulate granulopoiesis have not been determined. Our study identified potential NeuP-specific epigenetic regulators (Supplementary Fig. S6). One such regulator, EZH2 is a core component of PRC2, which acts as a H3K27 methyl-transferase. Proper function of the PRC2 complex is known to play an essential role from the early stage of development by controlling plasticity and differentiation of stem cells^85^. EZH2 has been suggested to be a positive regulator of hematopoiesis^86^. Expression of the gene encoding SUZ12, another component of the PRC2 complex, was also found to be NeuP-specific. Whether these are truly stage-specific epigenetic regulators that are crucial for the transition to neutrophils is a topic of great interest and a focus of our current research efforts. We also found that different members within an epigenetic family, such as HDAC, CHD, and KDM families, are expressed in a stage-specific manner, providing a model in which each member within a family plays a distinct role at a different stage of differentiation, despite the overall structural and functional similarities of family members.

Our purification of NeuP made it possible to more precisely track the behavior of neutrophil precursors in multiple types of emergency circumstances, including G-CSF and LPS stimulation, *Listeria* infection, and myeloid ablation by clodronate-liposomes. Our data revealed a common behavior of neutrophil precursors. NeuP cell numbers increased in most cases, but the proportion of proliferating cells significantly decreased, except under systemic infection condition. We suspect that the NeuP population is a heterogeneous mixture of myelocytes and metamyelocytes, given that micrographs showed two morphologically distinct cell types (Fig. 1d). Myelocytes are more proliferative than metamyelocytes^52^, and if the differentiation balance between the two populations is shifted toward metamyelocytes during demand-associated granulopoiesis, the proportion of proliferating NeuPs would decrease, which is in line with our observations. We examined whether morphological shift (ring- vs peanut-shaped nuclei) occurred in the NeuP populations under G-CSF stimulation, but did not observe any significant alteration in the ratio of the two types of NeuPs. (Supplementary Fig. S8). A recent study demonstrated that ROS production by bone marrow myeloid cells is critical for controlling emergency granulopoiesis through induction of the expansion of early progenitors such as GMPs^87^. Notably, we observed a trend toward an increase in NeuP cell numbers under most test conditions, the exception being systemic bacterial infection. Hence, it is conceivable that granulopoiesis triggered by infection or cytokines leads to an increase in NeuP cell numbers, probably due to increased input from precursor cells prior to the NeuP stage and acceleration of the differentiation process, leaving fewer non-proliferating cells in the NeuP stage. However, we did not observe any difference in cell numbers and EdU incorporation of GMP (Fig. 7a,b) in our settings. Rather, we observed overall accelerated differentiation of neutrophils during the demand-adaptive granulopoiesis (Fig. 7c), supporting the possibility that altered EdU incorporation may be caused by differential modulation of residence time at a certain stage(s) of neutrophil differentiation. When the acceleration occurs and which stage of neutrophil differentiation is a critical rate-determining step is not clear. This will be answered when a methodology to definitely sort out neutrophil precursors upstream of NeuPs is devised. Although speculative, it is possible that blood cell precursors must “choose” between differentiation and proliferation along the developmental path in response to various environmental cues, and this leads to a tipping of the balance toward differentiation, a dynamic response that we consider constitutes the hematopoietic or granulopoietic triage process.

In summary, we have devised a new method for isolating neutrophil precursors using the surface marker Ly6B. To our knowledge, this is the first report of a neutrophil precursor population that has been isolated at high purity by flow cytometry. This CD11b^+^Ly6G^lo^Ly6B^int^CD115^−^ NeuP population is proliferative and has the potential to differentiate into neutrophils *in vitro* and *in vivo*. In demand-adapted granulopoiesis, NeuPs increased in numbers and were skewed toward differentiation rather than proliferation. Gene expression profiling analyses suggested numerous factors that might be critical for neutrophil differentiation at the NeuP stage. Collectively, our findings provide insight that could lead to a deeper understanding of the control of granulopoiesis and may also help improve strategies for treating diseases caused by uncontrolled granulopoiesis or myeloablation. Notably, a recent report has shown that immature Ly6G^int^ cells with ring-shaped nuclei circulate in tumor-bearing mice and have tumor-promoting roles^88^. We suspect that these Ly6G^int^ cells are related to NeuPs, a possibility that we are currently exploring.

## Materials and Methods

### Mice

C57BL/6 J mice (gender and age-matched, 6–8 weeks) were purchased from Daehan Biolink (Korea). *Lyz2-cre* (*Lyz2*^*tm1*^(^*cre*^)^*Ifo*^) and *ROSA-eYFP* (*Gt*(*ROSA*)*26Sor*^*tm1*^(^*EYFP*^)^*Cos*^) were generous gifts from Drs. R.M. Locksley and C.A. Lowell (UCSF). S100A8-cre (*Tg*(*S100A8-cre,-EGFP*)*1Ilw/J*)^75^ were generously provided by Drs. I.L. Weissman (Stanford) and G.O. Ahn (Postech). For induction of demand-adapted granulopoiesis, 2.5 μg recombinant murine G-CSF (Peprotech), 20 μg lipopolysaccharide (LPS; Sigma), or 1 mg clodronate-liposomes (Clodronate Liposomes) was intravenously injected. Live *L. monocytogenes* 10403 S was injected into the tail vein and mouse footpads at 10^3^ and 10^4^ cfu per injection, respectively. All animal experiments were performed in accordance with guidelines and regulations for rodent experiments provided by the Institutional Animal Care and Use Committee (IACUC) of KAIST. The protocols for the study were approved by KAIST IACUC (KA2010-21, KA2014-09).

### Intra-BM transfer

Intra-BM transfer of sorted precursors was performed as described previously^89^. Briefly, after a small incision was made around the knee to visualize the kneecap, cells were transferred into the bone cavity with a syringe.

### Antibodies

Antibodies for flow cytometry were purchased from BD Biosciences, eBioscience, Biolegend, or Serotec, unless otherwise indicated. The antibodies used were CD3ε (clone 145-2C11 or 17A2), CD8α (53-6.7), CD11b (M1/70), CD11c (HL3), CD19 (1D3), CD24 (M1/69), CD45R (B220, RA3-6B2), CD44 (IM7), CD45.1 (A20), CD45.2 (104), CD49b (DX5), CD115 (AFS98), CD117 (2B8), CD133 (13A4), CD172 (p84), Class II (M5/114.15.2), FcεRIα (Mar-1), Gr-1 (RB6-8C5), Ki-67 (B56), Ly6B (7/4), Ly6C (HK1.4), Ly6G (1A8), NK1.1 (PK136), Siglec-F (E50-2440).

### Flow cytometry

BM cells were isolated from femur and tibia and dispersed into single-cell suspensions by passing through a 70-μm cell strainer (SPL, Korea). Erythrocytes were removed by ACK lysis. Cells were blocked with anti-CD16/32 and then stained for surface molecules. DAPI (4,6-diamidino-2-phenylindole; Roche) was used for dead cell exclusion. Live cells were counted with counting beads (Invitrogen). For intracellular staining, a Live/Dead Fixable Violet Dead-Cell Stain kit (Invitrogen) was used for dead cell exclusion. Cells were fixed with 3.7% formaldehyde and quenched with 10 mM glycine/phosphate-buffered saline (PBS). Fixed cells were permeabilized with 0.5% saponin/PBS and stained for intracellular molecules. Data were acquired on an LSRFortessa flow cytometer (BD Biosciences) and analyzed with FlowJo software (Tree Star).

### Sorting

Erythrocyte-depleted single-cell suspensions of BM cells, prepared as described above (flow cytometry), were blocked with anti-CD16/32. Whole BM suspensions were enriched for CD11b^+^ cells by staining with a biotin-conjugated anti-CD11b antibody followed by incubating with streptavidin-microbeads (Miltenyi) and magnetic separation. Enriched cells were stained for surface molecules, and dead cells were excluded by staining with DAPI. Sorting was performed on an Aria II or Aria III system (BD Biosciences) using an 85-μm nozzle.

### May-Grünwald-Giemsa staining

Cells were attached to glass slides using the Cytospin system (Thermo Scientific). Samples were air dried and then sequentially stained with May-Grünwald solution (Sigma-Aldrich) and Giemsa solution (Sigma-Aldrich). Images were captured with a Nikon Eclipse 80i equipped with a CCD camera DS-Ri1 (Nikon), and analyzed using Photoshop CS6 (Adobe).

### *In vitro* culture of NeuP or neutrophils

Sorted cells in RPMI (supplemented with 10% heat-inactivated FBS, 10 mM HEPES, 2 mM L-glutamine, 100 units/ml penicillin, 100 μg/ml streptomycin, and 50 μM β-mercaptoethanol) were cultured with or without congenic (CD45.1^+^) 10^6^ whole BM cells. G-CSF or GM-CSF (Peprotech) was added at 10 or 20 ng/ml, respectively.

### ROS production assay

NeuPs or neutrophils were either sorted from the BM or cultured *in vitro* in the presence of G-CSF for 24 hours at 37 °C. Cells were stained with 5 μM dihydrorhodamine 123 (Invitrogen). N-acetylcysteine (NAC) was treated while staining with DHR123 to show the negative staining. ROS production was stimulated by treating cells for 30 minutes with *E. coli* (DH5α, multiplicity of infection (MOI) = 10) alone for the sorted cells or with Low-Tox-M Rabbit Complement (Cedarlane) and *E. coli* (DH5α, MOI = 10) for the cultured cells. Dead cells were excluded with DAPI and stained cells were analyzed on an LSRFortessa (BD Biosciences); data were analyzed using FlowJo software (Tree Star).

### Phagocytosis analysis

NeuPs or neutrophils were either sorted from the BM or cultured *in vitro* in the presence of G-CSF for 24 hours at 37 °C. Sorted cells were incubated with FITC-conjugated microbeads (Polysciences, Fluoresbrite^TM^ Plain YG 0.5 micron microspheres, 0.46 μm) for 30 min at 37 °C. Cultured cells were stimulated with tdTomato-expressing *E. coli* (tdTomato-pcDNA3.1(+)-myc-His C, DH5α) for 30 minutes at a MOI of 10. Dead cells were excluded with DAPI and stained cells were on an LSRFortessa (BD Biosciences); data were analyzed using FlowJo software (Tree Star).

### RNA-seq

Total RNA was isolated using the RNeasy Mini kit and on-column DNase digestion (Qiagen). The sequencing library was prepared using the TruSeq RNA Sample Preparation kit v2 (Illumina). The Illumina multiplexing adapters were ligated to the cDNA, and the fragments were amplified by polymerase chain reaction (PCR). Sequencing was performed in paired-end 100-base-pair reads using a HiSeq 2500 system (Illumina).

### RT-PCR

Sorted NeuPs or neutrophils (10^6^ cells each) were homogenized in Tri-Reagent Solution (Ambion). RNA was extracted with chloroform, and precipitated with isopropanol and ethanol. DNA was removed by treatment with RQ1 DNase (Promega). cDNA was synthesized with Superscript III Reverse Transcriptase (Invitrogen). Gene expression was confirmed by PCR using the following primers which produce distinct amplicons on genomic and cDNA template.

- *Actb* forward: 5′-TCCAGCCTTCCTTCTTGGGT-3′,
- *Actb* reverse: 5′-GCACTGTGTTGGCATAGAGGT-3′
- *Mpo* forward: 5′-TCCCACTCAGCAAGGTCTT-3′,
- *Mpo* reverse: 5′-TAAGAGCAGGCAAATCCAG-3′,
- *Ltf* forward: 5′-ACCGCAGGCTGGAACATC-3′,
- *Ltf* reverse: 5′-CACCCTTCTCATCACCAATACAC-3′,
- *Mmp9* forward: 5′-ATAGAGGAAGCCCATTACAGG-3′,
- *Mmp9* reverse: 5′-GTGTACACCCACATTTGACG-3′

### EdU incorporation assay

For *in vivo* EdU incorporation assays, mice were intravenously injected with 0.5 mg EdU per mouse 2 hours before sacrifice. For *in vitro* EdU incorporation, sorted cells were cultured for 2 hours in the medium supplemented with 10 μM EdU. For dead cell exclusion, a Live/Dead Fixable Violet Dead-Cell Stain kit (Invitrogen) was used. Cells were fixed with 3.7% formaldehyde and quenched with 10 mM glycine/phosphate-buffered saline (PBS). Fixed cells were permeabilized with 0.5% saponin/PBS. EdU was stained with 10 μM azide-A488, 1 mM CuSO_4_, and 100 mM ascorbic acid in 0.5% saponin/PBS. Stained cells were analyzed on an LSRFortessa flow cytometer (BD Biosciences), and data were analyzed using FlowJo software (Tree Star).

## Additional Information

**How to cite this article**: Kim, M.-H. *et al*. A late-lineage murine neutrophil precursor population exhibits dynamic changes during demand-adapted granulopoiesis. *Sci. Rep.* **7**, 39804; doi: 10.1038/srep39804 (2017).

**Publisher's note:** Springer Nature remains neutral with regard to jurisdictional claims in published maps and institutional affiliations.

## Supplementary Material

Supplementary Information

Supplementary Table S1

Supplementary Table S2

## Figures and Tables

**Table 1:** Genes encoding transcription factors.

Cluster 1 NeuP & Neu	Cluster 2 NeuP	Cluster 3 Mono	Cluster 4 Mono & NeuP	Cluster 5 Mono & Neu	Cluster 6 Neu
*Bhlhe40*	*Cebpe*	*Atf6*	*Bcl11a*	*Camta2*	*Bcl6*
*E2f3*	*E2f7*	*Bach2*	*Mta1*	*Elk3*	*Carhsp1*
*Grhl1*	*Foxm1*	*Cebpa*	*Pias2*	*Fos*	*Creb3l3*
*Gtf2ird2*	*Foxp4*	*Fosb*	*Runx3*	*Fosl2*	*Ddit3*
*Hlf*	*Gfi1*	*Gtf3a*	*Satb2*	*Foxj2*	*Gli1*
*Hmgb2*	*Gtf2ird1*	*Jun*	*Smarcc1*	*Hbp1*	*Kdm5b*
*Jdp2*	*Hlx*	*Mta3*	*Snapc4*	*Hopx*	*Klf2*
*Lin28a*	*Hmgb1*	*Ncor2*	*Trerf1*	*Irf1*	*Litaf*
*Mxi1*	*Hmgb3*	*Nfxl1*	*Zbtb12*	*Irf5*	*Max*
*Nfe2*	*Lcorl*	*Nr2c1*	*Zbtb45*	*Irf9*	*Mier3*
*Pbx1*	*Mafg*	*Setdb2*	*Zfp27*	*Junb*	*Nfia*
*Pbx2*	*Mbd4*	*Stat2*	*Zfp362*	*Mitf*	*Pknox1*
*Satb1*	*Mxd3*	*Zfp229*	*Zfp653*	*Nr4a1*	*Preb*
*Stat4*	*Myb*	*Zfp316*	*Zmiz2*	*Ppard*	*Runx2*
*Zfp507*	*Nfyb*	*Zfp317*		*Rara*	*Tgif2*
*Zfp523*	*Ssrp1*	*Zfp59*		*Rarg*	*Zbtb39*
*Zscan2*	*Terf2*	*Zfp623*		*Relb*	*Zbtb42*
	*Tfdp2*	*Zfp64*		*Rxra*	*Zfp182*
	*Thap2*	*Zfp873*		*Tgif1*	*Zfp276*
	*Tshz1*	*Zkscan17*		*Zbtb4*	*Zfp654*
	*Wdhd1*	*Zscan20*		*Zfp467*	*Zfp691*
	*Whsc1*			*Zfp874b*	*Zfp719*
	*Zbtb14*				*Zfp740*
	*Zfp11*				*Zfp831*
	*Zfp111*				*Zhx2*
	*Zfp213*				*Zxdc*
	*Zfp324*				
	*Zfp367*				
	*Zfp473*				
	*Zfp930*				

A list of genes clustered in the transcription factor heat map shown in Fig. 4e. Cell types expressing high levels of genes within clusters are shown.
