# Supplementary material for: A late-lineage murine neutrophil precursor population exhibits dynamic changes during demand-adapted granulopoiesis
Source: Sci Rep. 2017 Jan 6;7:39804. doi: 10.1038/srep39804 (PMC5216372; doi:10.1038/srep39804)

Supplementary Information

for

A late-lineage murine neutrophil precursor population exhibits dynamic changes during demand-adapted granulopoiesis

Min-Hyeok Kim[1], Dongchan Yang[2], Mirang Kim[3], Seon-Young Kim[3], Dongsup Kim[2], and Suk-Jo Kang[1, *]

[1]Department of Biological Sciences, Korea Advanced Institute of Science and Technology, Daejeon, 34141, Republic of Korea

[2]Department of Bio and Brain Engineering, Korea Advanced Institute of Science and Technology, Daejeon, 34141, Republic of Korea

[3]Medical Genomics Research Center, Korea Research Institute of Bioscience and Biotechnology, Daejeon, 34141, Republic of Korea

Supplementary methods

Quality control, alignment, and calculation of expression level. An Agilent Bioanalyzer HS DNA assay was used for quality control of the cDNA library. The quality of RNA fragments was tested using SolexaQA[1]. Quality was assessed based on the fitness of the mean probability of error and the proportion of reads that satisfied cutoff criteria. Transcriptome analyses were then performed by applying Tuxedo suite[2] to FASTQ files whose quality was reliable. Paired-ended RNA-seq reads were aligned to the Mouse Genome Assembly (mm10) using TopHat[3]. The quality of Bam files from TopHat was evaluated using SAMStat [4]. The expression level of each gene was measured as FPKM (Fragments Per Kilobase of transcript per Million mapped reads) using Cufflinks[2].

Data filtering and normalization. To avoid misinterpretation due to false negative data generated by sequencing failure, genes with an FPKM value of zero were excluded from the analysis. The statistical distribution of gene expression was standardized by quantile normalization of data using the preprocessCore package (Affymetrix, Santa Clara, CA 95051, USA). After normalization, data were log-transformed for further analysis.

Gene expression profile analysis. The similarity of transcriptomes between cell populations was assessed by creating correlation heat maps and drawing scatter plots. The correlation heat map, based on Pearson correlation coefficients, between samples was drawn using the corrplot package (Renmin University of China, Beijing, People's Republic of China). Gene expression values for two cell types were plotted as a scatter plot using a graphics package (R Foundation for Statistical Computing, http://www.R-project.org). Here, genes with expression differences of 2-fold or greater were designated DEGs and plotted with cell type-specific colors. A heat map was drawn to display the transcriptome profile of each sample. DEGs were identified using Cuffdiff, which provides a statistical analysis of the indicated biological replicates. DEGs were defined as genes with a q-value (FDR from the Benjamini-Hochberg correction for multiple testing) less than 0.05. A heat map of the selected DEGs was drawn using MeV (http://www.tm4.org/); a heat map of transcription factors was also drawn. The list of mouse transcription factors was retrieved from AnimalTFDB[5]. Transcription factors with changes in expression greater than 3-fold between cell types were selected, and only genes that had less than a 2-fold difference between replicates were retained. A heat map was drawn with these

genes using the made4 package[6]. A principal component analysis (PCA) plot including all genes that passed quality control was drawn using prcomp and plot (R Foundation for Statistical Computing, http://www.R-project.org) and processed with data filtering and normalization. Principal components of transcriptomes were calculated using the stats package from the R Foundation for Statistical Computing (http://www.R-project.org).

Variations in expression during neutrophil differentiation. Microarray data from ImmGen[7] (www.immgen.org) was combined with RNA-seq data generated in the previous steps. CEL files for GMPs and neutrophils were downloaded from GEO using accession number GSE15907. These CEL files were read, and RMA (robust multi-array average)-normalized using the affy package in R (Center for Biological Sequence Analysis, Technical University of Denmark, Lyngby, Denmark)[8]. The differences in scales between microarray and RNA-seq data necessitated data conversion and normalization. Because microarray data follow a log-scale, RNA-seq data were log-transformed and a linear regression was performed to compare gene expression profiles of the two platforms. Gene expression profiles of GMPs and NeuPs were divided by that of neutrophils to calculate relative expression. The fold change in gene expression from GMPs to NeuPs was measured as

$$\frac{expr(\mathrm{NeuP})}{expr(\mathrm{GMP})} = \frac{\left(\frac{expr(\mathrm{Neu})}{expr(\mathrm{GMP})}\right)}{\left(\frac{expr(\mathrm{Neu})}{expr(\mathrm{NeuP})}\right)}$$

Genes were then categorized into groups based on the fold change of gene expression at two differentiation steps: GMP to NeuP ($\frac{expr(\mathrm{NeuP})}{expr(\mathrm{GMP})}$) and NeuP to Neutrophil ($\frac{expr(\mathrm{Neu})}{expr(\mathrm{NeuP})}$). If gene expression changed by 2-fold or more, genes were defined as up-regulated or down-regulated. Nine categories, including no change during differentiation, were assigned. A GO analysis was performed on the gene expression sets using DAVID (the Database for Annotation, Visualization and Integrated Discovery)[9].

Supplementary tables

Supplementary Table S1. Genes in expression profile groups.

ImmGen microarray data for neutrophils and GMPs and RNA-seq data for neutrophils and NeuPs were combined. DEGs were clustered into eight groups based on their expression profiles. Genes belonging to each group are listed.

Supplementary Table S2. GO term analysis of expression profile groups.

Genes in the expression profile groups were further analyzed for gene ontology using DAVID. GO terms with higher statistical significance are shown first, and genes belonging to each GO term are listed.

Supplementary Figures

Supplementary Figure S1. Monocyte contamination in neutrophil precursor analyses based on c-kit and Ly6G. (**a**) A flow cytometry analysis of neutrophil precursors based on expression of c-kit and Ly6G, performed as described previously[10]. (**b**) $CD115^+$ contaminating monocytes in each population are shown in histograms.

Supplementary Figure S2. Sorting scheme for murine bone marrow NeuPs. Murine bone marrow cells isolated from femurs and tibias were stained with antibodies. Dead cells (DAPI-incorporating) were excluded. Plot of FSC-A versus FSC-H; cells were gated for singlet cells. $CD11b^+$ cells negative for lineage ($CD3\varepsilon^-CD19^-NK1.1^-B220^-$) and CD115, and expressing an intermediate level of Ly6B were sorted by flow cytometry. The plot in the lower left shows a representative result demonstrating the purity of sorted cells.

Supplementary Figure S3. Size, granularity, and surface marker expression of NeuPs. (**a**) Size (FSC) and granularity (SSC) of NeuPs, neutrophils, monocytes and eosinophils, analyzed by flow cytometry. (**b**) Flow cytometry of surface markers for basophils (DX5 and Mar-1) and eosinophils (Siglec-F) on NeuPs in the BM, and basophils and eosinophils in the blood. (**c**) Flow cytometry of surface markers (CD24, CD44, and CD172) on NeuPs in the BM compared to neutrophils and monocytes in the BM. Data are representative of at least two independent experiments with two or more replicates.

Supplementary Figure S4. Procedure for combining ImmGen microarray data and RNA-seq data. GMP and neutrophil gene expression data from ImmGen project were combined with our RNA-seq DATA. Gene alignment was done using TopHat. Cufflinks was used to measure expression levels as FPKM values and transform the values to a logarithmic scale. Microarray data provided by ImmGen were processed using the affy package in R. Gene expression ratios between GMPs and neutrophils and between NeuPs and neutrophils were calculated, after which the ratios of the two ratios (GMPs/neutrophils and NeuPs/neutrophils) were calculated.

Supplementary Figure S5. Gene expression profile groups and GO terms. Genes were categorized based on fold changes in gene expression from GMPs to neutrophils via NeuPs; eight groups exhibiting changes between cell types are shown. Gene ontologies of genes within each group, based on DAVID, are shown. Data are combined from two to four independent

experiments for each cell type, with cells pooled from five to ten mice in each experiment.

Supplementary Figure S6. Epigenetic regulator genes in expression profile groups. DEGs in the expression profile groups encoding epigenetic regulators are listed according to their function, determined based on DAVID, AmiGO (Gene Ontology Consortium, http://amigo.geneontology.org/amigo), and QuickGO (EMBL-EBI, http://www.ebi.ac.uk/QuickGO). Known target sites and modifications are shown next to the gene name.

Supplementary Figure S7. Analysis of cell numbers during demand-adapted granulopoiesis. (**a**) Twenty-four hours prior to sacrifice, mice were injected with PBS or G-CSF (2.5 μg) (**b**) or were injected with PBS or LPS (20 μg). (**c**) Forty-eight hours before sacrifice, mice were injected with PBS (control) via the tail vein or with *Listeria* through footpads (10^4 cfu; local infection) or tail veins (10^3 cfu; systemic infection) (**d**) or were injected with PBS- or clodronate-liposomes (1 mg). Total cells and neutrophils in the bone marrows were counted. Depletion of monocytes in the blood by clodronate-liposomes, but not by PBS-liposomes, was confirmed. The significance of differences between stimulated and control groups was analyzed using Student's *t*-test. Data are representative of at least two independent experiments with two or more replicates (mean ± SD of N = 3 to 4 mice/group).

Supplementary Figure S8. Analysis of two morphologically distinct NeuP populations under emergency granulopoiesis. Cytological analysis of NeuPs isolated from the mice that were injected with PBS or G-CSF (2.5 μg) 24 hours prior to sacrifice. Nuclear morphology of isolated cells was analyzed with May-Grünwald-Giemsa staining and the ratio of cells containing either ring- or peanut-shaped nuclei was calculated (mean ± SD of N = 3 mice/group).

Supplementary Figure S9. The original full-length gel image of Fig. 4g. The original full-length gel image of Fig. 4g, showing RT-PCR analysis of granule marker genes (Mpo, Ltf, Mmp9) in NeuPs and neutrophils. M, a marker lane for DNA molecular weights.

References

1 Cox, M. P., Peterson, D. A. & Biggs, P. J. SolexaQA: At-a-glance quality assessment of Illumina second-generation sequencing data. *BMC bioinformatics* **11**, 485, doi:10.1186/1471-2105-11-485 (2010).

2 Trapnell, C. *et al.* Differential gene and transcript expression analysis of RNA-seq experiments with TopHat and Cufflinks. *Nature protocols* **7**, 562-578, doi:10.1038/nprot.2012.016 (2012).

3 Trapnell, C., Pachter, L. & Salzberg, S. L. TopHat: discovering splice junctions with RNA-Seq. *Bioinformatics* **25**, 1105-1111, doi:10.1093/bioinformatics/btp120 (2009).

4 Lassmann, T., Hayashizaki, Y. & Daub, C. O. SAMStat: monitoring biases in next generation sequencing data. *Bioinformatics* **27**, 130-131, doi:10.1093/bioinformatics/btq614 (2011).

5 Zhang, H. M. *et al.* AnimalTFDB: a comprehensive animal transcription factor database. *Nucleic acids research* **40**, D144-149, doi:10.1093/nar/gkr965 (2012).

6 Culhane, A. C., Thioulouse, J., Perriere, G. & Higgins, D. G. MADE4: an R package for multivariate analysis of gene expression data. *Bioinformatics* **21**, 2789-2790, doi:10.1093/bioinformatics/bti394 (2005).

7 Heng, T. S., Painter, M. W. & Immunological Genome Project, C. The Immunological Genome Project: networks of gene expression in immune cells. *Nature immunology* **9**, 1091-1094, doi:10.1038/ni1008-1091 (2008).

8 Gautier, L., Cope, L., Bolstad, B. M. & Irizarry, R. A. affy--analysis of Affymetrix GeneChip data at the probe level. *Bioinformatics* **20**, 307-315, doi:10.1093/bioinformatics/btg405 (2004).

9 Huang da, W., Sherman, B. T. & Lempicki, R. A. Systematic and integrative analysis of large gene lists using DAVID bioinformatics resources. *Nature protocols* **4**, 44-57, doi:10.1038/nprot.2008.211 (2009).

10 Satake, S. *et al.* C/EBPbeta is involved in the amplification of early granulocyte precursors during candidemia-induced "emergency" granulopoiesis. *Journal of immunology* **189**, 4546-4555, doi:10.4049/jimmunol.1103007 (2012).

11 Mitsuyama, M., Nomoto, K., Akeda, H. & Takeya, K. Enhanced elimination of Listeria monocytogenes at the site of delayed footpad reaction. *Infection and immunity* **30**, 1-4 (1980).

12 Pamer, E. G. Immune responses to Listeria monocytogenes. *Nature reviews. Immunology* **4**, 812-823, doi:10.1038/nri1461 (2004).

Figure S1

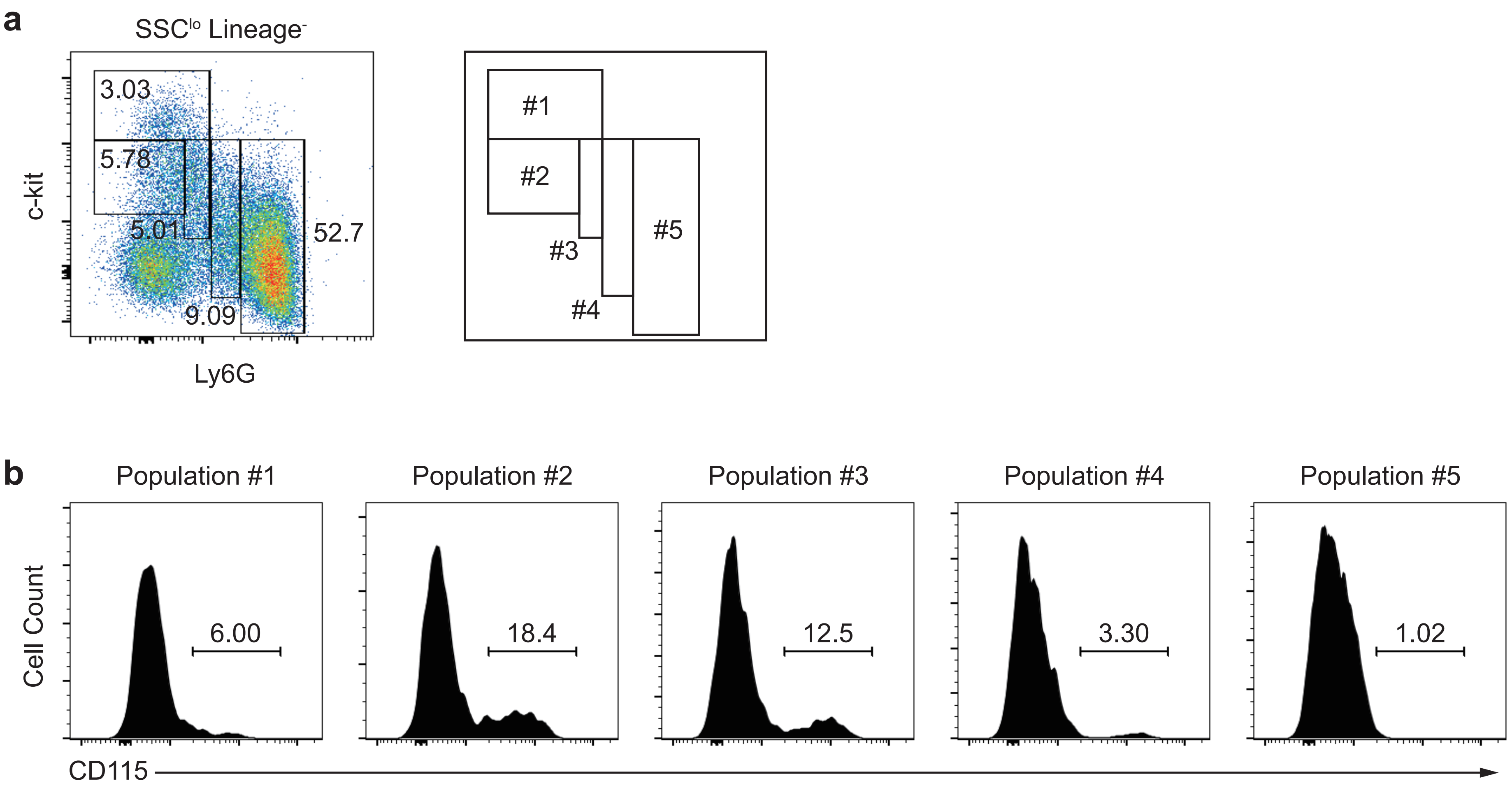
a
SSC^lo Lineage^-
3.03
5.78
5.01
52.7
9.09
c-kit
Ly6G
#1
#2
#3
#4
#5
b
Population #1
Population #2
Population #3
Population #4
Population #5
6.00
18.4
12.5
3.30
1.02
Cell Count
CD115

Figure S2

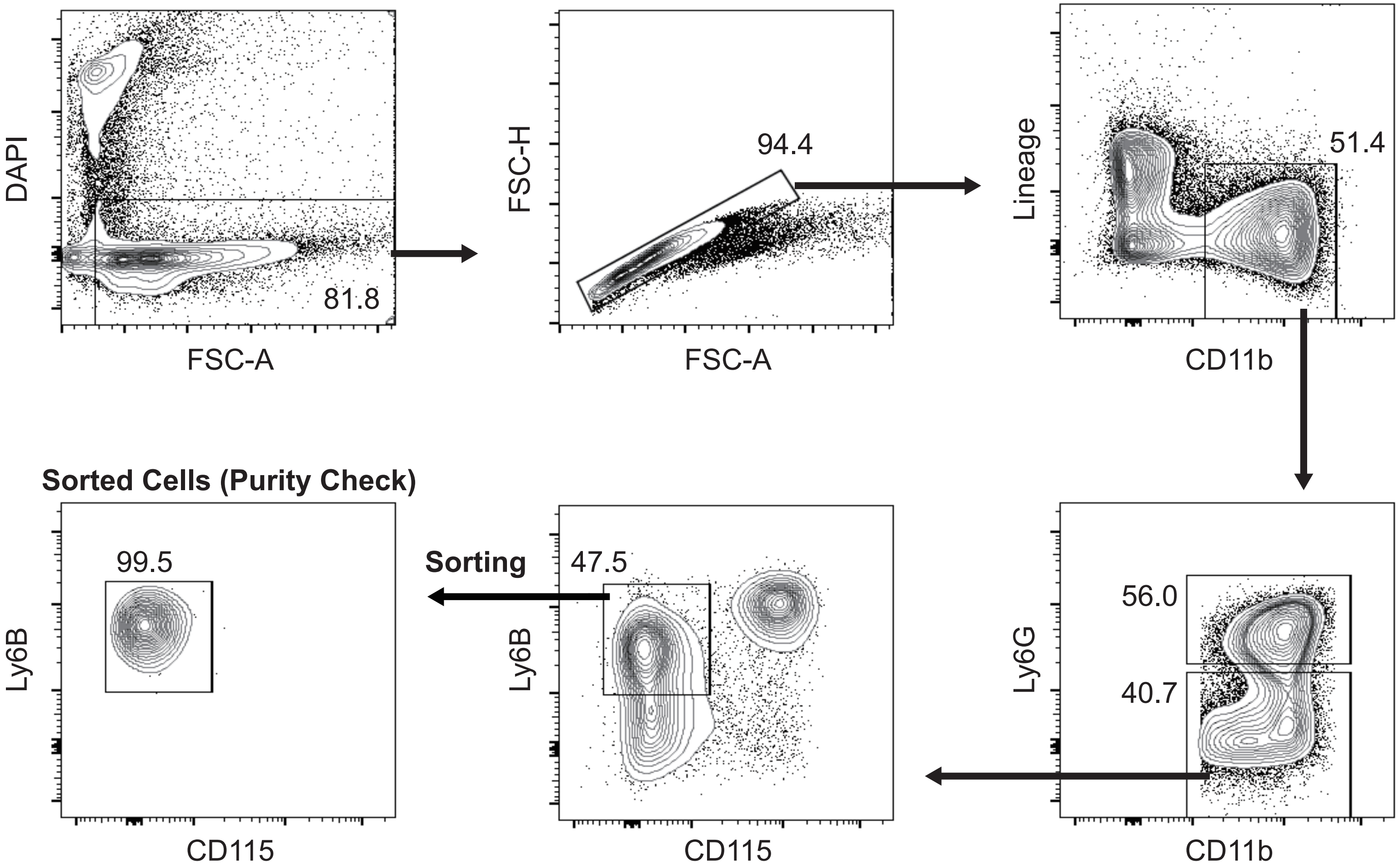

DAPI
FSC-A
81.8
FSC-H
FSC-A
94.4
Lineage
CD11b
51.4
Ly6G
CD11b
56.0
40.7
Ly6B
CD115
47.5
Sorting
Sorted Cells (Purity Check)
Ly6B
CD115
99.5

Figure S3

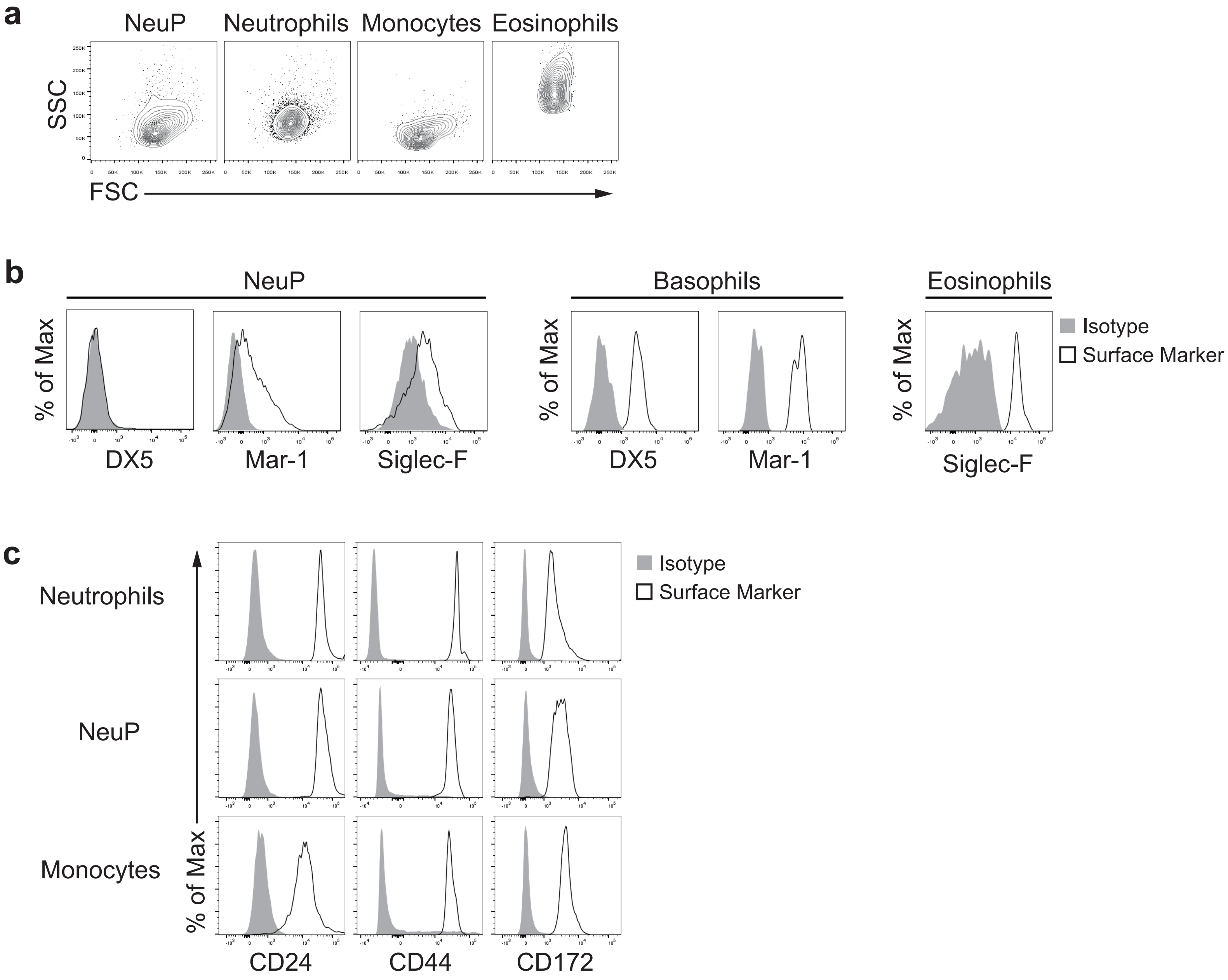

a
NeuP
Neutrophils
Monocytes
Eosinophils
SSC
FSC
b
NeuP
Basophils
Eosinophils
% of Max
DX5
Mar-1
Siglec-F
Isotype
Surface Marker
c
Neutrophils
NeuP
Monocytes
% of Max
CD24
CD44
CD172
Isotype
Surface Marker

Figure S4

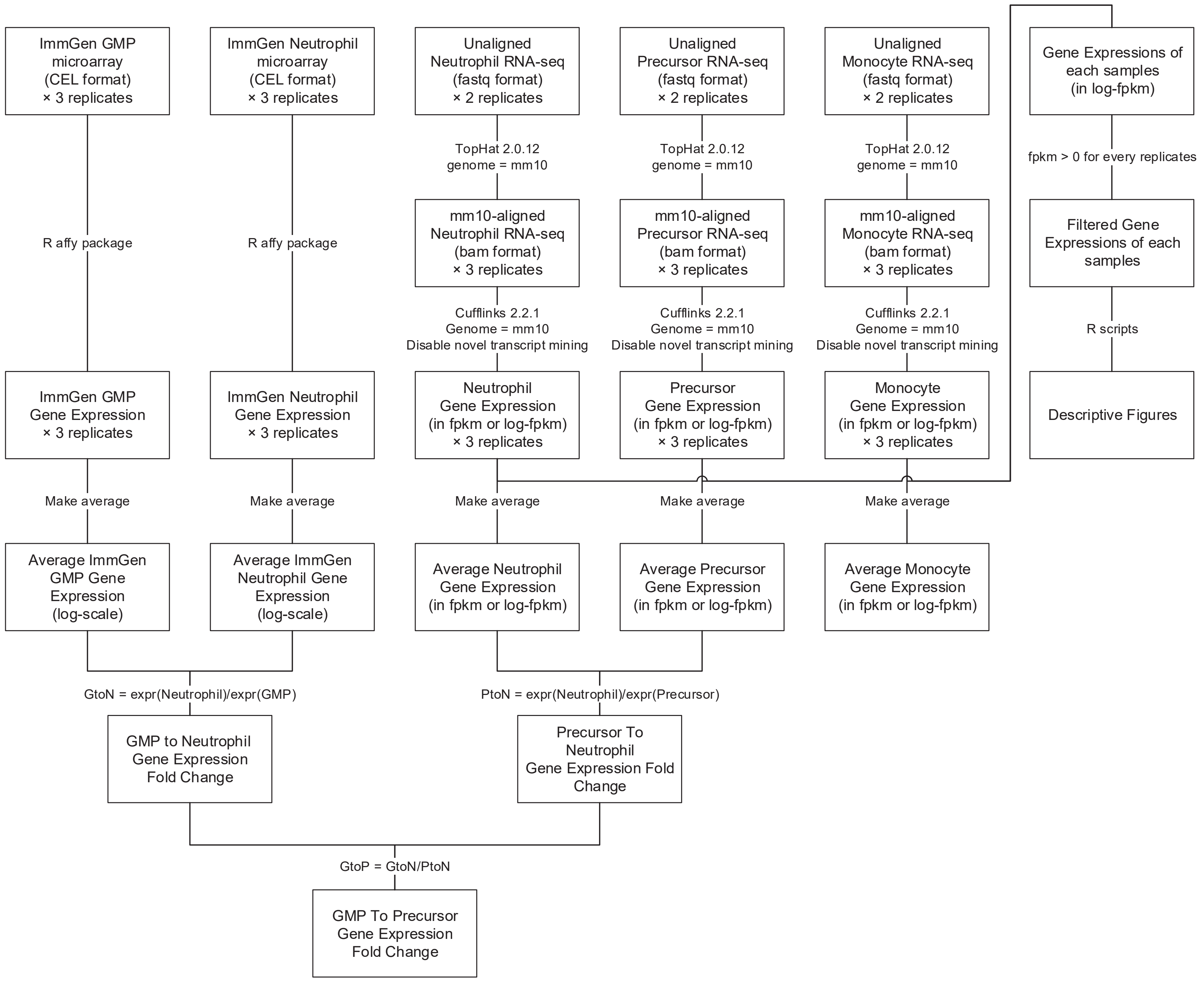
ImmGen GMP microarray (CEL format) × 3 replicates
ImmGen Neutrophil microarray (CEL format) × 3 replicates
Unaligned Neutrophil RNA-seq (fastq format) × 2 replicates
Unaligned Precursor RNA-seq (fastq format) × 2 replicates
Unaligned Monocyte RNA-seq (fastq format) × 2 replicates
Gene Expressions of each samples (in log-fpkm)
TopHat 2.0.12 genome = mm10
fpkm > 0 for every replicates
R affy package
mm10-aligned Neutrophil RNA-seq (bam format) × 3 replicates
mm10-aligned Precursor RNA-seq (bam format) × 3 replicates
mm10-aligned Monocyte RNA-seq (bam format) × 3 replicates
Filtered Gene Expressions of each samples
Cufflinks 2.2.1 Genome = mm10 Disable novel transcript mining
R scripts
ImmGen GMP Gene Expression × 3 replicates
ImmGen Neutrophil Gene Expression × 3 replicates
Neutrophil Gene Expression (in fpkm or log-fpkm) × 3 replicates
Precursor Gene Expression (in fpkm or log-fpkm) × 3 replicates
Monocyte Gene Expression (in fpkm or log-fpkm) × 3 replicates
Descriptive Figures
Make average
Average ImmGen GMP Gene Expression (log-scale)
Average ImmGen Neutrophil Gene Expression (log-scale)
Average Neutrophil Gene Expression (in fpkm or log-fpkm)
Average Precursor Gene Expression (in fpkm or log-fpkm)
Average Monocyte Gene Expression (in fpkm or log-fpkm)
GtoN = expr(Neutrophil)/expr(GMP)
PtoN = expr(Neutrophil)/expr(Precursor)
GMP to Neutrophil Gene Expression Fold Change
Precursor To Neutrophil Gene Expression Fold Change
GtoP = GtoN/PtoN
GMP To Precursor Gene Expression Fold Change

Figure S5

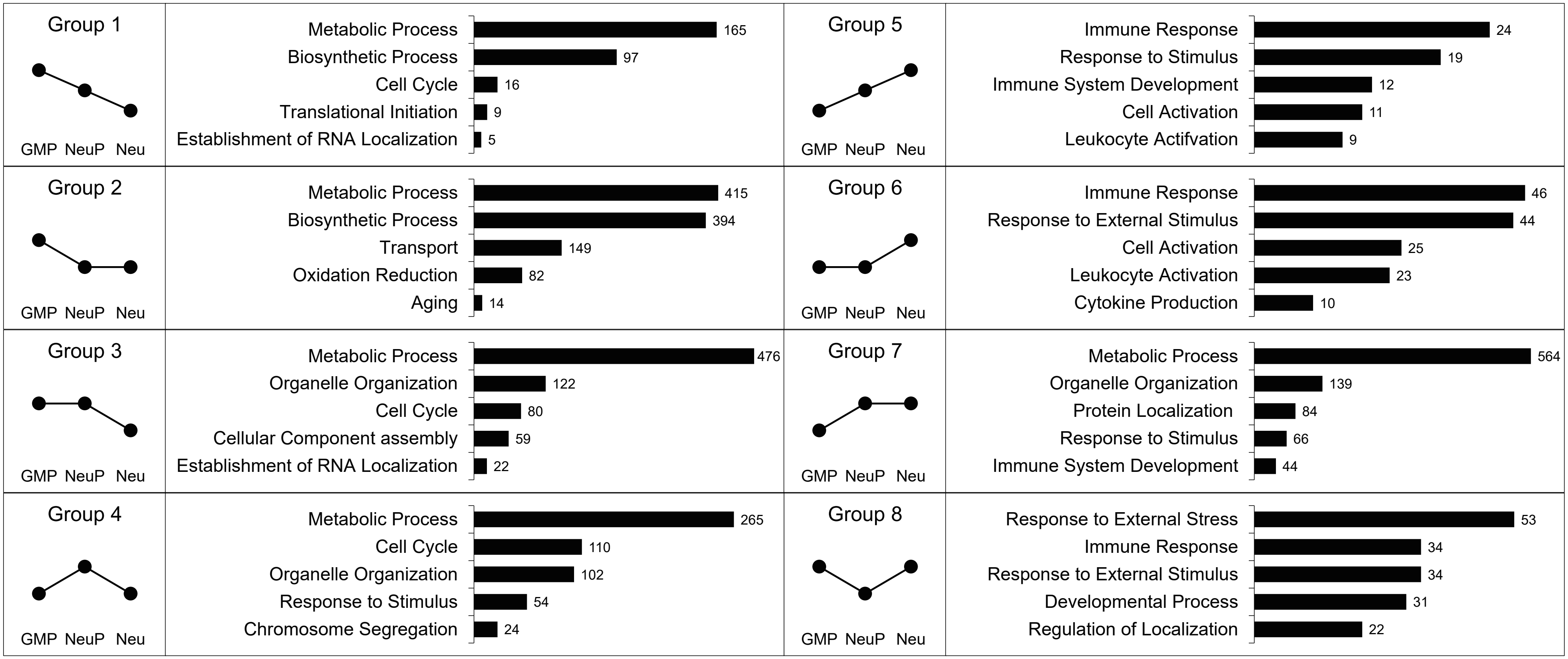
Group 1
GMP NeuP Neu
Metabolic Process 165
Biosynthetic Process 97
Cell Cycle 16
Translational Initiation 9
Establishment of RNA Localization 5
Group 5
GMP NeuP Neu
Immune Response 24
Response to Stimulus 19
Immune System Development 12
Cell Activation 11
Leukocyte Actifvation 9
Group 2
GMP NeuP Neu
Metabolic Process 415
Biosynthetic Process 394
Transport 149
Oxidation Reduction 82
Aging 14
Group 6
GMP NeuP Neu
Immune Response 46
Response to External Stimulus 44
Cell Activation 25
Leukocyte Activation 23
Cytokine Production 10
Group 3
GMP NeuP Neu
Metabolic Process 476
Organelle Organization 122
Cell Cycle 80
Cellular Component assembly 59
Establishment of RNA Localization 22
Group 7
GMP NeuP Neu
Metabolic Process 564
Organelle Organization 139
Protein Localization 84
Response to Stimulus 66
Immune System Development 44
Group 4
GMP NeuP Neu
Metabolic Process 265
Cell Cycle 110
Organelle Organization 102
Response to Stimulus 54
Chromosome Segregation 24
Group 8
GMP NeuP Neu
Response to External Stress 53
Immune Response 34
Response to External Stimulus 34
Developmental Process 31
Regulation of Localization 22

Figure S6

	Chromatin organization/modification						
	Histone modification					Chromatin remodeling	Others
	Histone acetylation	Histone deacetylation	Histone methylation	Histone demethylation	Others		
**G1**	***Kat2a*** H3K14, H4K12<br>***Ruvbl2*** H2A, H4<br>***Ruvbl1*** H2A, H4		***Ehmt2*** H3K9, H3K27<br>***Prmt7***				
**G2**	***Actl6a*** H2A, H4<br>***Bmi1***<br>***Csrp2bp*** H3<br>***Hat1***<br>***Mbd3***<br>***Meaf6*** H2A, H3K14, H4K5, H4K8, H4K12, H4K16	***Hdac2*** H3K9, H3K14, H3K18, H4K16<br>***Hdac6*** H3K9, H3K14, H3K18, H4K16<br>***Hdac7*** H3K9, H3K14, H3K18, H4K16<br>***Hdac8*** H3K9, H3K14, H3K18, H4K16<br>***Rbm14***	***Dnmt1*** H3K4, H3K9<br>***Prmt6*** H2AR3, H3R2, H4R3<br>***Suv39h1*** H3K19	***Kdm2b***	***Brcc3*** H2A K63 Ub<br>***Huwe1*** Ub	***Chd1l***<br>***Chd9***<br>***Rbbp4***<br>***Rbbp7***<br>***Smarcb1***<br>***Smarcc1***	***Asf1a***<br>***Cbx3***<br>***Eny2***<br>***H2afx***<br>***Hira***<br>***Mcm2***<br>***Smarcal1***
**G4**	***Phf17*** H3, H4K5, H4K8, H4K12, H4K16	***Rcor1*** H4	***Ezh2*** H3K27<br>***Suz12*** H3K27<br>***Whsc1***		***Baz1b*** P<br>***Gsg2*** H3T3 P<br>***Rnf168*** Ub	***Pbrm1***	***Aebp2***<br>***Asf1b***<br>***Fam175a***<br>***Hells***<br>***Nasp***<br>***Rbl1***
**G6**	***Ing4*** H3, H4K5, H4K8, H4K12, H4K16		***Ezh1*** H3K27	***Kdm5b*** H3K4	***Ube2b*** H2A Ub		***Phf1***<br>***Phf21a***
**G7**	***Brd8*** H2A, H4<br>***Crebbp***<br>***Ep300*** H2B, H4<br>***Epc1*** H2A, H4<br>***Kat2b*** H3<br>***Taf1***	***Hdac4*** H3K9, H3K14, H3K18, H4K16<br>***Tbl1xr1***	***Ash1l***<br>***Baz2a*** H3K9, H4K20, H4 DeAc<br>***Mll1*** H3K4, H4K16 Ac<br>***Mll3*** H3K4<br>***Mll5***<br>***Nsd1***<br>***Rtf1*** H3K4<br>***Setd1b***<br>***Setd2*** H3K36<br>***Setd8***<br>***Suv420h1*** H4K20<br>***Whsc1l1***	***Jmjd1c***<br>***Kdm3a***<br>***Kdm4c*** H3K9<br>***Sirt1*** H3K9	***Mysm1***<br>***Rnf20*** H2B Ub<br>***Uimc1*** Ub	***Baz1a***<br>***Chd7***<br>***Chd8***<br>***Ttf1***	***2210018M11rik***<br>***Bcorl1***<br>***Ctcf***<br>***Epc2***<br>***Hltf***<br>***Hnrpll***<br>***Ikzf1***<br>***Kdm5a***<br>***Ncor1***<br>***Nr3c1***<br>***Rb1***<br>***Rbl2***<br>***Shprh***<br>***Tlk1***<br>***Tlk2***

Figure S7

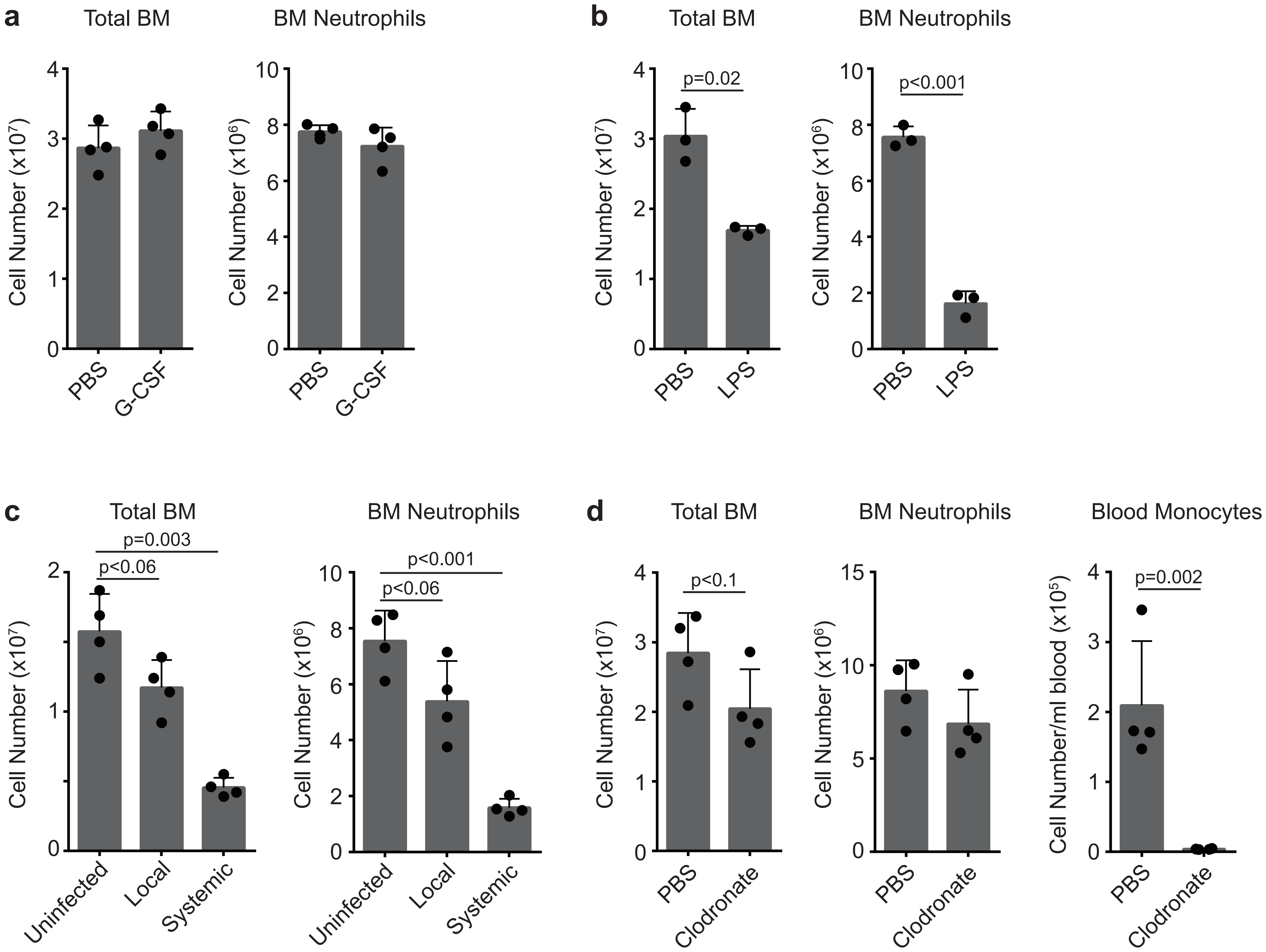

a
Total BM
BM Neutrophils
Cell Number (x10⁷)
Cell Number (x10⁶)
PBS
G-CSF
b
Total BM
BM Neutrophils
p=0.02
p<0.001
PBS
LPS
c
Total BM
BM Neutrophils
p=0.003
p<0.06
p<0.001
Uninfected
Local
Systemic
d
Total BM
BM Neutrophils
Blood Monocytes
p<0.1
p=0.002
Cell Number/ml blood (x10⁵)
PBS
Clodronate

Figure S8

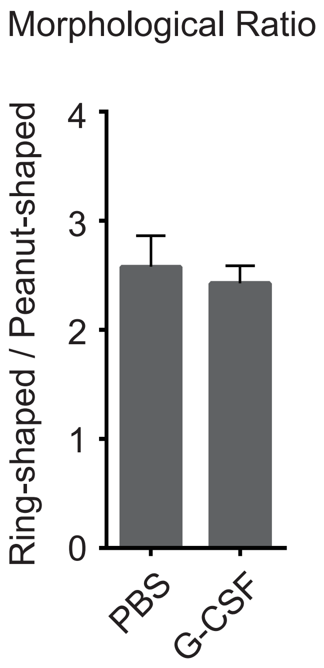

Morphological Ratio
Ring-shaped / Peanut-shaped
4
3
2
1
0
PBS
G-CSF

Figure S9

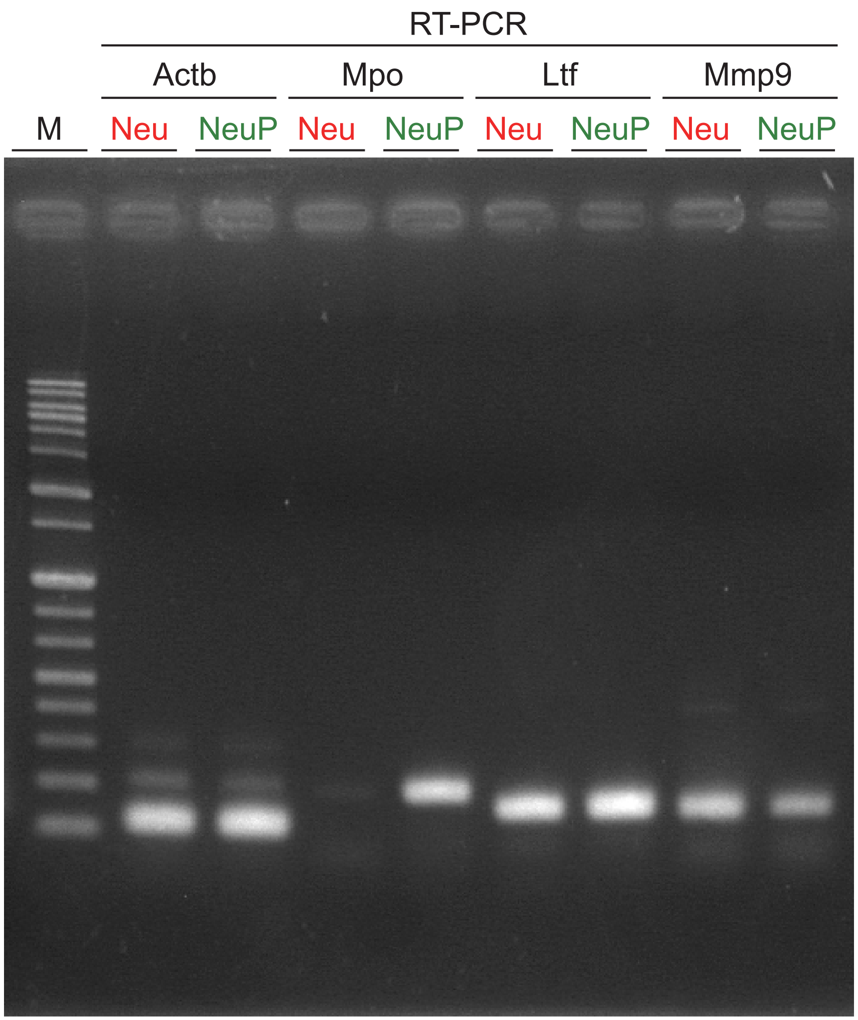

Supplement: Supplementary Information [file srep39804-s1.pdf]
